# A growth-coupled progesterone-responsive biosensor for high-throughput microfluidic screening in *Saccharomyces cerevisiae*

**DOI:** 10.1016/j.synbio.2025.12.004

**Published:** 2026-01-02

**Authors:** Yucheng Hu, Jinde Chen, Shaofang Tian, Yang Zhang, Zhiqian Zhang, Ao Jiang, Yi-Rui Wu, Baoshun Zhang

**Affiliations:** a College of Pharmaceutical Sciences, Southwest University, Chongqing, 400715, China; b Tidetron Bioworks Technology (Guangzhou) Co., Ltd., Guangzhou Qianxiang Bioworks Co., Ltd., Guangzhou, 510000, China

**Keywords:** Progesterone-responsive biosensor, Growth-coupled selection, ARTP mutagenesis, Fluorescence-activated droplet sorting, *Saccharomyces cerevisiae*, Steroid biotransformation

## Abstract

Poor aqueous solubility of steroid precursors, such as pregnenolone and progesterone, limits microbial biotransformation and high-throughput strain screening, representing a bottleneck for strain improvement and potential industrial applications.

To address this, we developed a growth-coupled progesterone-responsive biosensor in *Saccharomyces cerevisiae*, integrated with a hydroxypropyl-β-cyclodextrin (HP-β-CD) system to enhance intracellular steroid availability. The biosensor links progesterone formation to cell growth and fluorescence, with selection stringency finely tuned via an IPTG-inducible lac operator and 3-aminotriazole (3-AT) to suppress low-producing cells. Coupled with atmospheric and room temperature plasma (ARTP) mutagenesis, the growth-coupled biosensor–FADS platform identified five yeast variants capable of improved conversion of pregnenolone to progesterone while expressing 3β-hydroxysteroid dehydrogenase (3β-HSD) without altering the enzyme itself. The progesterone production of these selected variants was subsequently validated using 1 mM pregnenolone as the substrate, showing 2.0–3.37-fold higher titers than the wild-type strain, demonstrating proof-of-concept. Microfluidic droplet encapsulation allowed clear separation of high-producers, highlighting the platform's selectivity, robustness, and scalability. This synthetic biology–driven system integration platform provides a practical, modular, and high-throughput strategy for screening poorly water-soluble steroid-producing yeast. It is adaptable to other bioactive molecules, can support future enzyme evolution, and demonstrates potential for broader biotechnological applications.

## Graphical abstract

**Figure:**
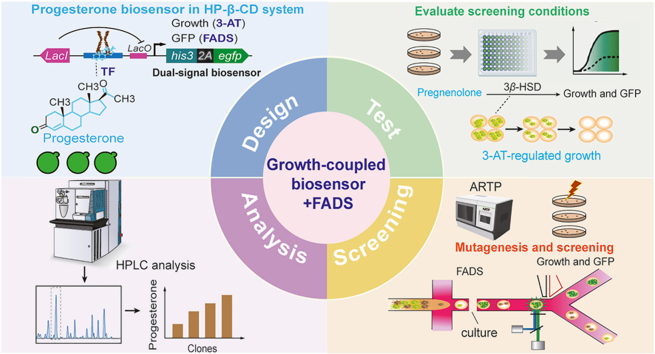

## 1 Introduction

Microbial production of high-value chemicals is a central application of synthetic biology, enabling the programmable engineering of metabolic pathways for pharmaceuticals, hormones, and other bioactive molecules [[1], [2], [3]]. Despite significant progress, efficiently identifying high-performing strains remains challenging, particularly for metabolites with low aqueous solubility or multi-step biosynthetic pathways [4]. Such limitations not only reduce the catalytic efficiency of key enzymes but also constrain substrate uptake and impede high-throughput evaluation of engineered strains. Moreover, the complex interplay between pathway enzymes, cofactor availability, and host metabolism can generate unintended bottlenecks, highlighting the need for system-level strategies that integrate both genetic and environmental factors to optimize cellular performance [5]. Advances in evolutionary engineering and high-throughput screening provide tools to systematically explore microbial genetic diversity, although the complexity of metabolic and regulatory networks often exceeds current screening capabilities [6,7]. In particular, integrating transcription factor (TF)-based biosensors with droplet microfluidics or other high-throughput screening technologies enables rapid and quantitative assessment of large mutant libraries, accelerating strain optimization and chassis engineering [8].

Steroid hormones such as pregnenolone and progesterone are widely used intermediates in pharmaceutical synthesis and biomedical research. Their hydrophobic nature creates intrinsic challenges for microbial biosynthesis, including inefficient cellular transport, suboptimal enzymatic conversion, and poor compatibility with conventional high-throughput screening methods. In progesterone biosynthesis, 3β-HSD catalyzes the rate-limiting conversion of pregnenolone to progesterone, emphasizing the necessity of optimizing both enzymatic activity and host performance [9,10]. Furthermore, the low aqueous solubility of steroid precursors limits intracellular availability, motivating the use of solubilizing agents such as HP-β-CD to enhance substrate uptake and improve microbial conversion efficiency. Balancing metabolic flux and cofactor availability in the host chassis is also critical to overcoming bottlenecks in multi-step pathways.

High-throughput screening strategies that link metabolite production to measurable phenotypes are essential for strain optimization. Conventional methods, including colorimetric assays or growth-based selection, often provide limited throughput and quantitative resolution [[11], [12], [13], [14]], making them insufficient for screening low-solubility substrates or complex metabolic pathways. TF-based biosensors provide a versatile alternative by converting intracellular metabolite levels into detectable outputs such as fluorescence or growth, and have been successfully integrated with evolutionary engineering to enhance metabolic flux and enzyme performance [15,16]. However, hydrophobic steroid substrates present additional challenges, including low cellular uptake, inefficient ligand binding, and high background signals, which reduce biosensor reliability and screening efficiency. Fluorescence-only screening strategies further face limitations such as host autofluorescence, leaky reporter expression, photobleaching, and false-positive signals arising from sensor decoupling or mutations in the reporter module. These issues are particularly pronounced in microfluidic droplets, where small volume fluctuations can reduce discrimination between high and low producers. In contrast, growth-coupled screening strategies provide an inherent fitness-based linkage between metabolite production and cell proliferation, enabling selective enrichment of productive variants while penalizing non- or low-producing cells [17]. By directly tying metabolite formation to host fitness, growth-coupled selection enhances robustness, minimizes false positives, and supports stringent, scalable selection in microdroplet environments. Successful TF-based biosensor–FADS strategies for other metabolites, such as naringin in *S. cerevisiae* [18], methylglyoxal in *E. coli* [19], and cis,*cis*-muconic acid in *S. cerevisiae* [20], demonstrate the potential of combining biosensing with microfluidic screening to efficiently select high-performing chassis strains. Despite the demonstrated potential of progesterone-responsive TFs for environmental monitoring [21,22], their application in directed evolution for steroid production remains underdeveloped, highlighting a clear opportunity for high-throughput strain engineering.

**Fig. 1:**
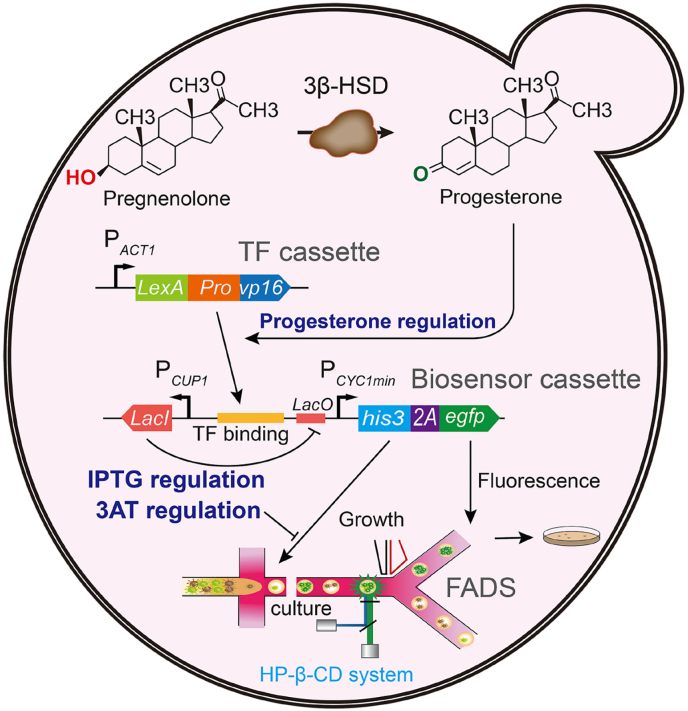
Schematic illustration of a FADS platform based on a growth-coupled progesterone-responsive biosensor in *S. cerevisiae*. The figure depicts the biosensor working mechanism, single-cell droplet encapsulation, and fluorescence-activated droplet sorting of encapsulated cells.

To address these challenges, we constructed a modular, growth-coupled progesterone-responsive biosensor in *S. cerevisiae* and integrated it with a FADS platform for high-throughput strain selection (Fig. 1). The biosensor employs a dual-reporter system, co-expressing EGFP for fluorescence readout and HIS3 for growth-based selection via a self-cleaving 2A peptide. Progesterone produced from pregnenolone by 3β-HSD activates a synthetic LexA–PRO–VP16 transcription factor, coupling metabolite formation to measurable outputs. IPTG induction and 3-AT supplementation allow tuning of selection stringency, while ARTP mutagenesis introduces genetic diversity into the host chassis. Encapsulation of single yeast cells in microfluidic droplets enables FADS to efficiently enrich variants displaying both fluorescence and growth, allowing quantitative evaluation of high-performing chassis strains.

Overall, this growth-coupled biosensor–FADS platform provides a robust, modular, and scalable system for high-throughput screening of poorly water-soluble steroid-producing yeast. By integrating substrate solubilization, genetic diversification, and dual-mode screening, the platform enables precise selection of optimized chassis strains. Beyond progesterone, this system can be adapted to other steroidal or bioactive molecules with appropriate optimization, providing a versatile framework for enzyme engineering, pathway development, and broader synthetic biology applications.

## 2 Materials and methods

### 2.1 Strains and culture conditions

All *S. cerevisiae* strains and plasmids used are listed in Tables S1 and S2. Yeast strains were derived from the CEN.PK2-1c background and cultured in YPD or SD dropout media (Coolaber, China) for plasmid selection. *E. coli* DH5α (TransGen) was used for plasmid propagation in LB medium at 37 °C with appropriate antibiotics. HP-β-CD (4 % w/v) was added where indicated to improve steroid solubility. Progesterone and pregnenolone were purchased from Aladdin and Macklin (China). Stock solutions of 3-AT (2.5 M) and IPTG (1 M) were obtained from Coolaber (China). PCR was performed using 2 × Phanta Flash Master Mix (Vazyme), and plasmids were assembled with ClonExpress Ultra One Step Cloning Kit V3 (Vazyme) following the manufacturers’ protocols.

### 2.2 Plasmid construction and strain engineering

The human *3β-HSD* gene (Gene ID: 3284), codon-optimized and synthesized by GenScript, was cloned under the TEF1 promoter and ADH1 terminator in a CEN/ARS plasmid (HY-E163). The plasmid was transformed into S077 to generate strain S081. A synthetic transcription factor, LexA-pro-VP16, comprising the bacterial LexA DNA-binding domain, human progesterone receptor ligand-binding domain, and VP16 activation domain, was expressed under the ACT1 promoter (HY-E125). This construct was integrated into the *ura3* locus of S064 via CRISPR/Cas9 to yield S065.

A reporter cassette containing a minimal CYC1 promoter with four LexA binding sites fused to EGFP was constructed in plasmid HY-E001 [23]. To enable IPTG-inducible regulation and reduce basal expression, LacO sites were inserted into the CYC1 promoter, and LacI was expressed under a weak copper-inducible CUP1 promoter [24], generating plasmids HY-E164, HY-E165, and HY-E166. Reporter plasmids HY-E090, HY-E164, HY-E165, and HY-E166 were integrated into the *leu2* locus of S065 to generate strains S067, S068, S077, S078, and S079. Yeast transformation was performed using the lithium acetate/single-stranded carrier DNA/PEG method [25].

### 2.3 HP-β-CD effects on steroid solubility, growth, and biosensor output in *S. cerevisiae*

The solubilization capacity of HP-β-CD for pregnenolone and progesterone was assessed by adding 1 mg of each steroid to 1 mL of SD medium containing 1–10 % (w/v) HP-β-CD, with or without 2 % (v/v) DMSO. The mixtures were vortexed, filtered through a 0.22 μm membrane, and analyzed by HPLC.

The physiological effects of HP-β-CD on strain S077 were evaluated by culturing single colonies in 5 mL SD/-Leu medium at 30 °C and 250 rpm for 48 h. Cells were harvested, washed twice with sterile physiological saline, and inoculated into fresh SD/-Leu medium containing 0 %, 1 %, 2 %, 4 %, or 10 % (w/v) HP-β-CD. Two control groups were included: one with 2 % (v/v) DMSO and one without DMSO. Cultures were adjusted to an initial OD_600_ of ∼0.08, transferred to 96-well microplates (100 μL per well), and incubated at 30 °C with continuous shaking [26]. OD_600_ was recorded hourly for 48 h using a Tecan Spark microplate reader.

To evaluate the effect of HP-β-CD on biosensor output, strain S077 harboring an EGFP-based progesterone-responsive biosensor was cultured in SD/-Leu medium supplemented with 0.2 mM progesterone and 1–4 % (w/v) HP-β-CD. After incubation at 30 °C and 250 rpm for 12–14 h, cells were collected, washed, and resuspended in PBS to an OD_600_ of 1.0. EGFP fluorescence was measured and normalized to OD_600_ using a Tecan Spark microplate reader.

### 2.4 Characterization of a progesterone-responsive EGFP biosensor in *S. cerevisiae*

Strain S077 was streaked onto SD/-Leu agar plates, and single colonies were used to inoculate 5 mL of SD/-Leu liquid medium. Cultures were incubated at 30 °C with shaking at 250 rpm until the optical density at 600 nm reached 1.5–2.0. Cells were harvested by centrifugation at 3000×*g* for 3 min, washed twice with sterile physiological saline, and resuspended in fresh SD/-Leu medium supplemented with 4 % (w/v) HP-β-CD for subsequent assays.

For induction experiments, cell suspensions were diluted to an initial OD_600_ of 0.20, and inducers were added at final concentrations of 0–250 μM progesterone and 0–100 mM IPTG. Progesterone stock solutions (50 mM in DMSO) and IPTG stock solutions (1 M in water) were prepared in advance. The final DMSO concentration in all media was maintained at 2 % (v/v) to ensure consistent inducer permeability.

Cells were distributed into 24-well microplates (500 μL per well, n = 3) and incubated at 30 °C with orbital shaking at 250 rpm for 12–14 h using an LSE digital microplate shaker. After incubation, cells were washed and resuspended in PBS to an OD_600_ of 1.0. Bulk EGFP fluorescence and OD_600_ were measured using a Tecan Spark microplate reader. Fluorescence intensities were normalized to OD_600_ to account for variations in cell density.

### 2.5 3-AT–Regulated growth-coupled progesterone biosensor

Single colonies of strains S077 and S081 were picked from selection plates and inoculated into SD/-Leu or SD/-Leu/-Ura liquid media, respectively. Cultures were incubated at 30 °C with shaking at 250 rpm for 48 h. Cells were harvested by centrifugation at 3000 rpm for 5 min, washed twice with sterile physiological saline, and resuspended in fresh medium.

Strain S077 was inoculated into SD/-Leu/-His medium supplemented with 4 % (w/v) HP-β-CD, progesterone (0–100 μM), IPTG (0 or 1 mM), and 3-AT (0–30 mM). Strain S081 was cultured in SD/-Leu/-Ura/-His medium containing 4 % HP-β-CD, pregnenolone (150, 300, or 900 μM), IPTG (0 or 1 mM), and 3-AT (0–10 mM). Cultures were aliquoted into 96-well microplates (100 μL per well) at an initial OD_600_ of ∼0.06. Growth was monitored via OD_600_ using a Tecan SPARK microplate reader at 30 °C with continuous orbital shaking at 1440 rpm.

The effect of HP-β-CD on 3β-HSD activity was evaluated using strain S081. Cells were cultivated in SD/-Leu/-Ura medium containing 4 % HP-β-CD and pregnenolone at final concentrations of 100, 300, or 900 μM. Cultures were incubated at 30 °C with shaking at 250 rpm, and samples were collected every 24 h. Cells were lysed using an automated tissue grinder, and supernatants were filtered through 0.22 μm membranes prior to HPLC analysis. Progesterone concentrations were normalized to OD_600_ to assess catalytic efficiency.

### 2.6 FADS for high-throughput screening

The fluorescence-activated droplet sorting (FADS) setup was built on an inverted microscope (IX73, Olympus) mounted on a vibration-isolated platform. Excitation was provided by a 20 mW, 488 nm solid-state laser with heatsink [8]. Fluorescence emission was detected by a photomultiplier tube (PMT), and signals were processed using a custom LabVIEW program. Droplet sorting was triggered by high-voltage pulses (1 kV, 30 kHz) generated by a Trek amplifier. Images of droplet formation and sorting were captured using a high-speed camera (Phantom Miro, Vision Research).

Microfluidic chips were fabricated from PDMS using standard soft lithography. Yeast cells (initial OD_600_ = 0.3) were suspended in SD/-Leu/-Ura/-His medium containing 4 % HP-β-CD, 1 mM IPTG, 3 mM 3-AT, and 900 μM pregnenolone. The cell suspension was loaded into a syringe and infused into a single-emulsifier device at 1.5 μL/min, while fluorinated oil (HFE-7500 with 5 % surfactant) flowed as the continuous phase at 5 μL/min. Monodisperse droplets were generated at a 25 μm × 30 μm × 30 μm flow-focusing junction. Single-cell encapsulation efficiency was evaluated microscopically in three independent experiments, showing ∼80 % of droplets containing exactly one cell, consistent with a Poisson distribution [27].

Generated droplets were collected in sterile tubes and incubated at 30 °C for 48–60 h. After incubation, the droplets were reinjected into a sorting chip (50 μm depth, 65 μm width) at a flow rate of 0.1 μL/min, while a sheath flow of fluorinated oil was applied at 5 μL/min to hydrodynamically focus the droplets at the junction. High-fluorescence droplets exceeding a predefined threshold were selectively sorted. High-fluorescence droplets were sorted at a throughput of approximately 120 droplets per second. The enrichment of high-producing droplets was verified by random microscopic observation.

### 2.7 HPLC analysis of progesterone and pregnenolone

Yeast samples were lysed using a Tissuelyser. The lysates were centrifuged, and the resulting supernatants were filtered through 0.22 μm membranes prior to HPLC analysis. Progesterone and pregnenolone concentrations were determined following the protocols described in the Chinese Pharmacopoeia.

Chromatographic separation was performed on a Sinochrom C8300a column (250 × 4.6 mm, 5 μm; Elite Instruments, China) using a methanol–acetonitrile–water (35:25:40, v/v/v) mobile phase. Detection wavelengths were set at 241 nm for progesterone and 210 nm for pregnenolone. The column temperature was maintained at 28 °C, with a flow rate of 1.0 mL/min and an injection volume of 10 μL. All analyses were conducted in triplicate to ensure reproducibility.

### 2.8 Statistical analysis

All experiments were performed in biological triplicates unless otherwise stated. Each biological replicate was measured in technical triplicates, and the mean of technical replicates was used for subsequent analysis. Data are presented as mean ± standard deviation (SD) from three independent biological replicates (n = 3).

Dose–response curves were fitted using a four-parameter logistic (4 PL) model. Half-maximal inhibitory concentrations (IC_50_) and Hill coefficients were reported with 95 % confidence intervals determined by profile likelihood. Curve fitting and data visualization were conducted using GraphPad Prism 10.1.1.

Group differences were assessed by one-way analysis of variance (ANOVA), followed by Sidak's multiple comparisons test for selected pairs, with p-values adjusted to control for type I error. Normality and homogeneity of variance were confirmed using the Shapiro–Wilk test and Levene's test, respectively. Statistical significance was defined as *p* < 0.05.

## 3 Results and discussion

### 3.1 Development and comparative evaluation of growth-coupled progesterone biosensors in *S. cerevisiae*

To identify an optimized progesterone-responsive configuration for quantitative and tunable sensing, five biosensor constructs (S067, S068, S077, S078, and S079) were systematically evaluated (Fig. 3A). Fluorescence responses across a progesterone gradient (0–250 μM) were fitted using a 4 PL model to determine key performance parameters (Table S4). The baseline biosensor S067 consisted of a synthetic progesterone-responsive transcription factor controlling an EGFP reporter, whereas S068 introduced a self-cleaving 2A peptide to co-express HIS3 and EGFP, enabling parallel growth and fluorescence readouts [29]. Both S067 and S068 displayed broad dynamic spans (25,807 a.u. and 28,797 a.u., respectively) with strong curve fits (R^2^ > 0.99). However, high basal fluorescence (510–669 a.u.) compromised sensitivity at low progesterone concentrations (<5 μM) and precluded external regulation, making them less suitable for tunable sensing applications.

To enable externally controlled repression, LacI–LacO regulatory modules were integrated into the dual-reporter design, generating the engineered variants S077 (1 × LacO), S078 (2 × LacO), and S079 (3 × LacO). LacI was expressed under the weak, copper-inducible CUP1 promoter, and its binding to LacO sites repressed EGFP and HIS3 expression. The low basal activity of CUP1 ensured minimal background fluorescence [24,30]. As shown in Fig. 3B and summarized in Table S4, S077 exhibited minimal basal fluorescence and a low threshold of ∼5 μM and achieved approximately fourfold induction between 0 and 100 μM progesterone (Span = 1468 a.u.; Bottom = 477.1 a.u.; R^2^ = 0.991; HillSlope = 2.045). In contrast, increasing LacO copy number in S078 and S079 progressively strengthened repression but reduced dynamic range (794.3 a.u. and 479.3 a.u., respectively) increased threshold (∼10 μM) and curve stability. For S079, over-repression flattened the dose–response curve, preventing reliable HillSlope estimation and reducing quantitative precision. These results indicate that excessive LacI occupancy can overly constrain promoter activation, emphasizing the importance of balanced regulatory strength in biosensor optimization.

The tunable performance of LacI-regulated sensors was further assessed by IPTG-induced derepression at a fixed progesterone concentration (0.2 mM). Fluorescence responses to IPTG (0–100 mM) revealed dose-dependent derepression across engineered strains (Fig. 3C). Quantitative analysis based on 4 PL curve fitting shows that S077 displayed the broadest and most linear tunable range, with Top = 18,801 a.u., Bottom = 2019 a.u., Span ≈16,782 a.u., and SNR ≈9.3. In comparison, S078 and S079 exhibited narrower adjustable spans (S078: Top = 6431 a.u.; Bottom = 1101 a.u.; Span ≈ 5330 a.u.; SNR ≈ 5.8; S079: Top = 1984 a.u.; Bottom = 729 a.u.; Span ≈ 1255 a.u.; SNR ≈ 2.7). These results indicate that increasing LacO copy number reduces the operable range and dynamic response, flattening the induction curve and compromising tunability. The near-linear increase of fluorescence with IPTG concentration in S077 demonstrates that LacI repression can be quantitatively relieved, providing predictable, externally tunable control suitable for gradient-based microfluidic screening.

Overall, introducing LacI-based regulation transformed the biosensor from a static reporter into a dynamically adjustable sensing module. Among all configurations, S077 achieved the optimal balance between low background, robust induction, high SNR, wide tunable range, and a low threshold (∼5 μM) under IPTG-uninduced conditions, allowing sensitive detection at low progesterone concentrations. These integrated characteristics justify the selection of S077 as the foundation for subsequent high-throughput screening and provide a versatile regulatory framework for growth-coupled, quantitatively tunable biosensing and microfluidic evolution of steroid-producing yeast.

### 3.2 Tunable EGFP expression in *S. cerevisiae* S077 under IPTG control

The engineered strain S077 was evaluated for EGFP activation across a progesterone concentration range of 0–100 μM under varying IPTG levels (0–100 mM) to determine optimal conditions for downstream growth-coupled biosensor applications (Fig. 4A–D). Fluorescence dose–response curves were fitted using a 4 PL model to quantify Bottom, Top, Span, IC_50_, and HillSlope parameters (Table S5).

Increasing IPTG concentrations progressively elevated maximal fluorescence (Top) and expanded the dynamic range (Span), demonstrating tunable EGFP expression. At 0 mM IPTG, basal fluorescence remained low (Bottom = 476.9 a.u.), with a Span of 1467 a.u. and IC_50_ = 24.32 μM, providing a clear quantitative detection window. Both Top and Span increased steadily with IPTG up to 30 mM, reaching a maximal Span of ∼17,400 a.u., whereas additional IPTG beyond this point produced minimal further activation, consistent with near-complete LacI derepression. Compared to 0 mM IPTG, the Span at 30 mM IPTG increased approximately 12-fold, highlighting the strong tunable control afforded by the LacI–IPTG system.

Linear correlations between progesterone concentration and EGFP fluorescence were observed across all IPTG levels within the 0–100 μM range (Fig. 4C). Even without IPTG, the correlation remained statistically significant (R^2^ = 0.8381, *p* < 0.0001), and low IPTG levels (1 mM) further improved linearity (R^2^ = 0.8811, *p* < 0.0001). These results indicate a moderate-to-strong, progesterone-dependent fluorescence response, sufficient for high-throughput microfluidic screening. Low IPTG levels maintained robust signals while limiting overexpression and background fluorescence, thereby enabling reliable, quantitative assessment of progesterone production within a tunable biosensor framework.

Autofluorescence measured in the absence of progesterone under SD/-Leu medium supplemented with 4 % HP-β-CD and 2 % DMSO was negligible at IPTG ≤1 mM. Higher IPTG levels (>1 mM) increased autofluorescence up to ∼1.6-fold of baseline at 30–100 mM IPTG (Fig. 4D), likely due to elevated leaky expression of HIS3, co-expressed with EGFP in the progesterone-responsive system. Excessive HIS3 expression can enhance basal transcriptional activity, increasing background fluorescence and potentially interfering with 3-AT–mediated growth selection [23].

For growth-coupled biosensor applications, basal fluorescence remained low at 0–1 mM IPTG, while the dynamic range allowed sufficient induction. The fluorescence Span increased from 1467 a.u. at 0 mM IPTG to 2472 a.u. at 1 mM IPTG, demonstrating controlled tunability without overexpression. Collectively, these results indicate that 0–1 mM IPTG achieves an optimal balance between inducible EGFP activation and background suppression, providing a robust, tunable platform for quantitative progesterone monitoring and microfluidic high-throughput screening.

### 3.3 Optimal HP-β-CD concentration in yeast cultures

**Fig. 2:**
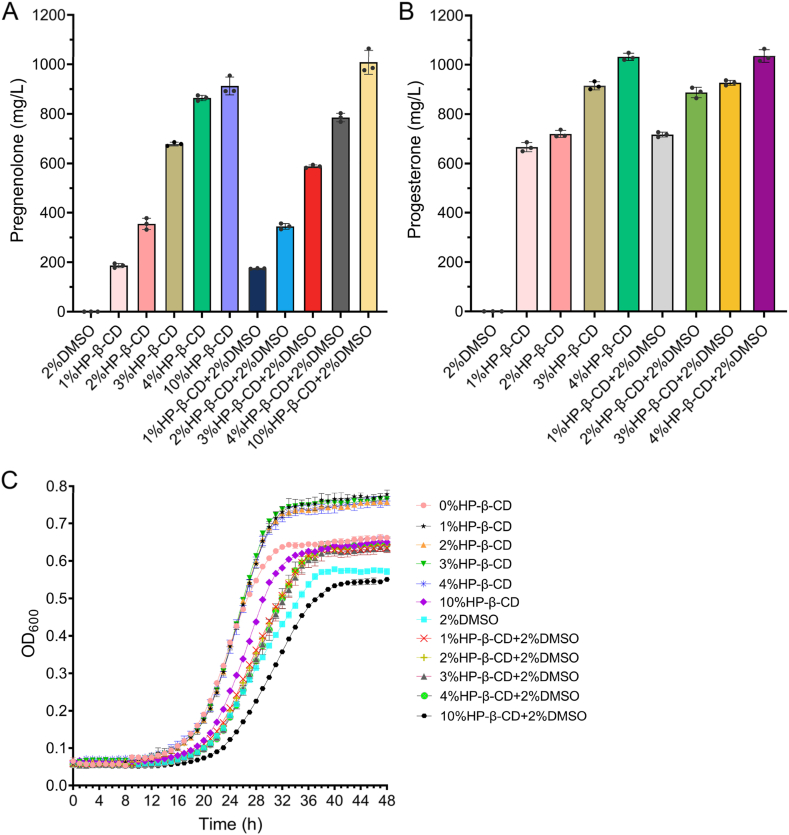
Effects of HP-β-CD on steroid solubility and *S. cerevisiae* growth. (A) Solubility of 1 mg pregnenolone in 1 mL SD medium supplemented with 1–10 % (w/v) HP-β-CD, with or without 2 % (v/v) DMSO, quantified by HPLC. (B) Solubility of 1 mg progesterone in 1 mL SD medium supplemented with 1–4 % (w/v) HP-β-CD, with or without 2 % (v/v) DMSO, quantified by HPLC. (C) Growth curves of strain S077 cultured in SD medium lacking leucine (SD/–Leu) with varying HP-β-CD concentrations (0–10 % w/v), with or without 2 % (v/v) DMSO.

Progesterone and pregnenolone are steroidal compounds with poor aqueous solubility due to their highly hydrophobic structures, limiting their bioavailability in microbial production systems. To overcome this limitation and enhance host performance, we evaluated HP-β-CD as a solubilizing agent [28]. Solubility assays showed that pregnenolone solubility increased with HP-β-CD concentration, reaching 0.86 mg/mL at 4 % (w/v) HP-β-CD and plateauing at 0.91 mg/mL at 10 % (w/v) (Fig. 2A). Similarly, progesterone solubility improved with increasing HP-β-CD concentration, achieving complete dissolution of 1 mg/mL at 4 % HP-β-CD (Fig. 2B). The presence of 2 % DMSO slightly decreased pregnenolone solubility while slightly increasing that of progesterone, but these minor effects did not significantly alter the solubilizing efficiency of HP-β-CD for either steroid. These results indicate that HP-β-CD substantially alleviates solubility limitations, thereby enhancing the availability of these steroids for microbial metabolism.

**Fig. 3:**
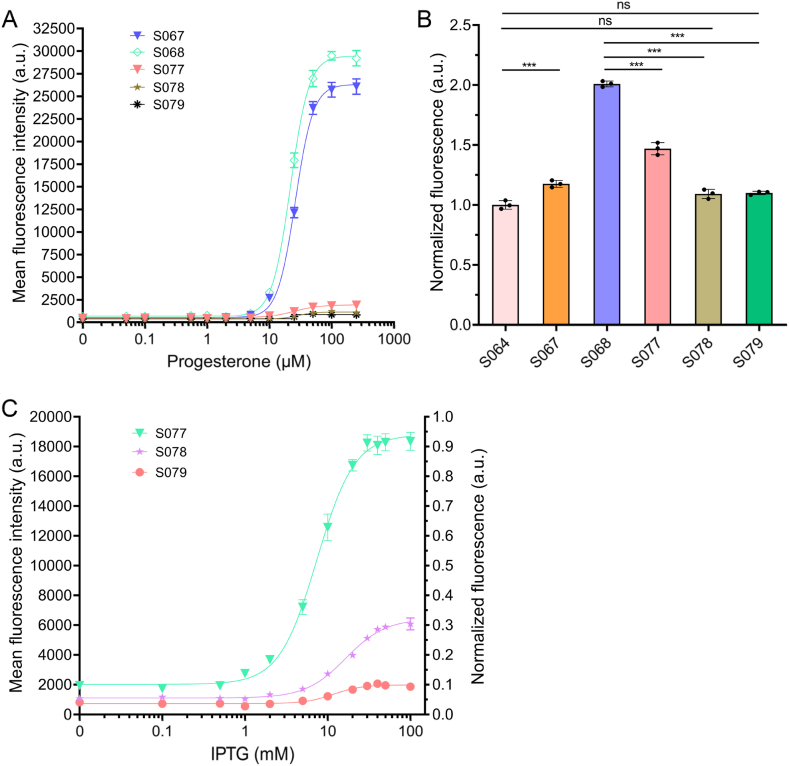
Fluorescence-based evaluation of engineered progesterone-responsive biosensors in *S. cerevisiae*. (A) Dose–response fluorescence of S067, S068, S077, S078, and S079 across 0–250 μM progesterone. (B) Basal fluorescence comparison of wild-type S064 and biosensor strains (∗*p* < 0.05, ∗∗*p* < 0.01, ∗∗∗*p* < 0.001). (C) IPTG-dependent fluorescence induction of S077 (1 × LacO), S078 (2 × LacO), and S079 (3 × LacO) at 200 μM progesterone.

**Fig. 4:**
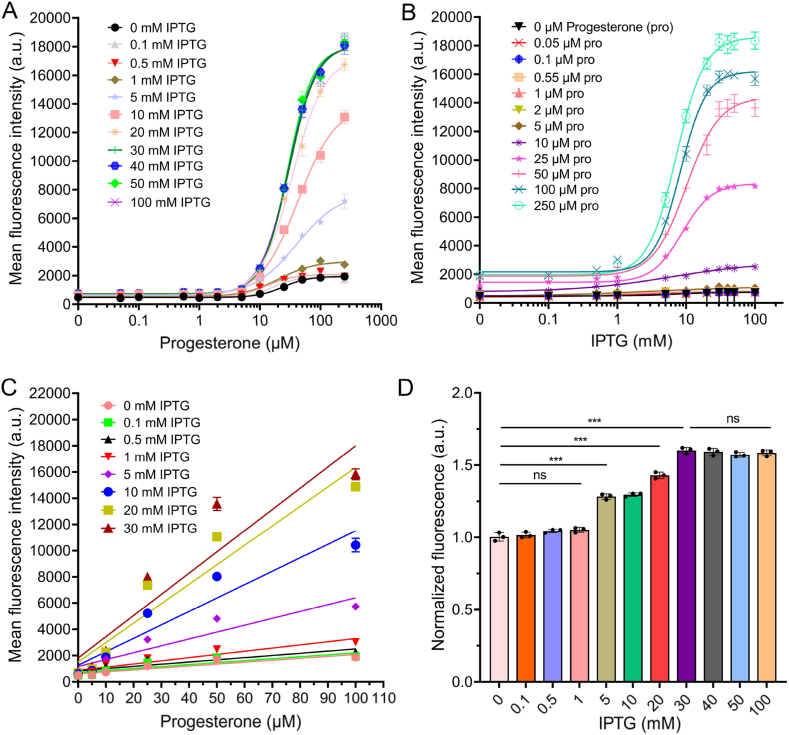
Fluorescence-based evaluation of the progesterone-responsive biosensor in S077 strain. (A–B) Dose–response curves of the dual-input promoter under varying progesterone and IPTG concentrations. EGFP induction can be tuned by adjusting either inducer. Fluorescence intensity was measured at OD600 = 1. (C) Linear regression of fluorescence response within 0–1 mM IPTG and 0–100 μM progesterone, showing strong correlation. (D) Autofluorescence of the biosensor in the absence of progesterone under 0–100 mM IPTG. Significance levels: ∗*p* < 0.05, ∗∗*p* < 0.01, ∗∗∗*p* < 0.001.

Growth assays revealed that 1–4 % HP-β-CD significantly promoted *S. cerevisiae* growth compared to the control without HP-β-CD, particularly in the absence of DMSO (Fig. 2C). In contrast, 10 % HP-β-CD mildly inhibited growth. Addition of 2 % DMSO alone slightly reduced growth, but co-supplementation with 1–4 % HP-β-CD partially alleviated this effect. Notably, the combination of 2 % DMSO and 10 % HP-β-CD caused pronounced growth inhibition, likely due to altered membrane permeability at high additive concentrations.

To evaluate the effect of HP-β-CD on biosensor output, the S077 strain harboring an EGFP-based progesterone-responsive biosensor was cultured with 0.2 mM progesterone under varying HP-β-CD concentrations ranging from 1 % to 4 %. No fluorescence was detected in the absence of progesterone, confirming the specificity of the biosensor response (Fig. S1A). Fluorescence measurements showed a positive correlation between HP-β-CD concentration and EGFP expression, with fluorescence at 4 % HP-β-CD approximately double that observed at 1 % HP-β-CD (Fig. S1B).

Collectively, these results identify 4 % HP-β-CD as the optimal concentration, balancing improved steroid solubility, yeast growth compatibility, and maximal biosensor responsiveness. This optimal concentration provides essential support for the subsequent development of a biosensor-based high-throughput screening platform, enhancing the applicability of steroid compounds in microbial systems.

### 3.4 Validation of the growth-coupled biosensor responsive to progesterone

To validate the specificity of the progesterone-responsive, growth-coupled biosensor in S077, fluorescence responses were measured in the presence of pregnenolone or structurally related steroid compounds. S077 lacking 3β-HSD was treated with pregnenolone at final concentrations of 0, 150, 300, and 900 μM (Fig. S2A), or with the five structurally related steroid compounds at 100 μM each (Fig. S2B). Fluorescence intensities were normalized to the respective no-compound control for each experiment, and no significant changes were observed (n = 3, *p* > 0.05), indicating that none of the compounds significantly activated the biosensor within the tested concentration range. These results establish a baseline for subsequent experiments using S081, in which pregnenolone is enzymatically converted to progesterone, enabling activation of the biosensor and growth-coupled detection.

**Fig. 5:**
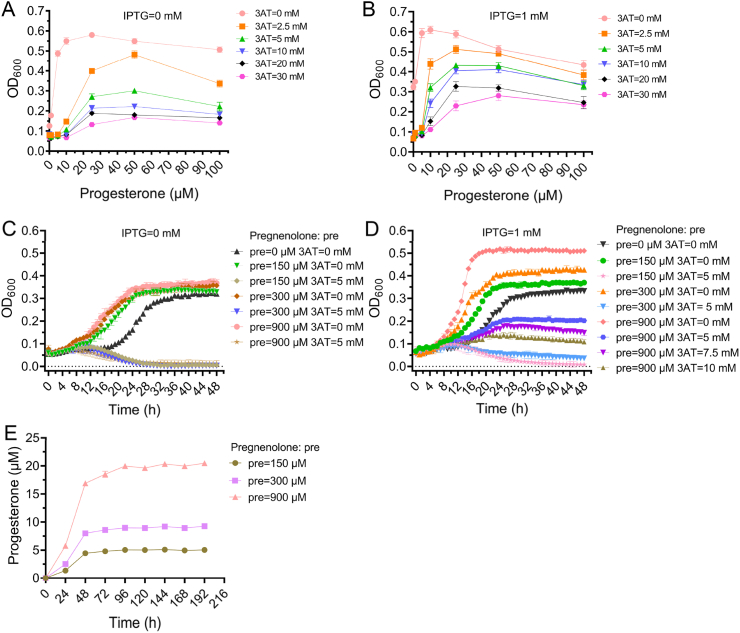
Validation of the growth-coupled progesterone-responsive biosensor under 3-AT regulation. (A, B) Growth curves of strain S077 cultured for 36 h in SD medium with 4 % HP-β-CD, lacking leucine and histidine, under 3-AT selection, with 0 mM (A) or 1 mM (B) IPTG, exposed to 0–100 μM progesterone. (C, D) Growth curves of strain S081 cultured in SD medium with 4 % HP-β-CD, lacking leucine, histidine, and uracil, with pregnenolone at 150, 300, and 900 μM, under 3-AT selection, with 0 mM (C) or 1 mM (D) IPTG. (E) Biotransformation of pregnenolone to progesterone by S081 in SD medium with 4 % HP-β-CD, lacking leucine and uracil, with a final DMSO concentration of 2 % (v/v). Cultures were grown for 192 h, and progesterone concentration was quantified every 24 h.

To minimize background growth and reduce false positives, 3-AT, a competitive HIS3 inhibitor, was employed [31,32]. S077 exhibited basal growth even in the absence of progesterone, consistent with low-level HIS3 expression. The addition of 2.5 mM 3-AT effectively suppressed this background growth, enhancing sensor specificity. In 3-AT-free conditions, S077 growth increased with progesterone concentration, reaching a peak OD_600_ at 25 μM; higher progesterone concentrations reduced growth, likely due to cytotoxicity [33]. Notably, these experiments were performed at very low initial cell density (OD_600_ ≈ 0.06), resembling sparse microfluidic droplet conditions, where direct exposure to high progesterone levels amplifies toxicity (Fig. 5A and B).

Building on these baselines, S081 growth under pregnenolone was evaluated. At initial OD_600_ ≈ 0.06, pregnenolone is gradually converted to progesterone via 3β-HSD, allowing slower local accumulation and reduced cytotoxic effects, thereby enabling reliable growth-coupled detection. Growth curves under varying pregnenolone and 3-AT concentrations indicated that 3-AT ≥5 mM effectively suppressed background growth, ensuring sensor specificity (Fig. 5C and D). IPTG induction further promoted HIS3 expression, accelerating cell proliferation.

Biotransformation assays revealed maximal progesterone titers of 5.01, 9.08, and 20.08 μM under 150, 300, and 900 μM pregnenolone, respectively, all within the sensor's dynamic detection range (Fig. 5E). All cultures were shaken; the highest titer (20.08 μM) was achieved after 96 h, whereas microfluidic assays operate over shorter timescales, demonstrating the sensor's suitability for high-throughput screening.

Together, these results establish a pregnenolone-indirectly responsive, growth-coupled biosensor using S081, consistent with steroid-dependent yeast growth phenotypes. Importantly, the system links product formation to a quantifiable growth output, enabling sensitive and specific detection of progesterone in microdroplet-based high-throughput assays, and providing a robust platform for directed evolution of steroid biotransformation pathways in *S. cerevisiae*.

### 3.5 Screening and characterization of *S. cerevisiae* mutants via a progesterone-responsive biosensor and FADS

FADS was employed using microfluidic chips to generate uniform droplets, each acting as an isolated microreactor physically separated by the surrounding oil phase to prevent cross-contamination. This configuration allowed precise selection of mutants based on product-coupled fluorescence signals through the growth-coupled progesterone-responsive biosensor [[34], [35], [36]]. To establish a high-throughput screening platform, 3-AT concentrations were optimized to suppress spontaneous growth of S081. Experiments were performed in SD medium supplemented with 4 % HP-β-CD, lacking leucine, uracil, and histidine, and containing 900 μM pregnenolone. IPTG (1 mM) significantly enhanced S081 growth after 60 h at 30 °C, likely via induction of the Lac operon and upregulation of histidine biosynthesis. Increasing 3-AT progressively inhibited growth, with 3 mM effectively suppressing spontaneous growth, establishing the optimal screening threshold (Fig. S3). Microscopy confirmed that most droplets contained single cells, and droplet shrinkage due to metabolic activity likely increased local reporter concentration, supporting accurate high-throughput sorting [37,38]. Notably, droplet screening results differed from those in 96-well plate cultures (Fig. 5C and D), likely due to higher initial cell densities and faster growth rates in the plate setup, which required increased 3-AT to achieve comparable growth inhibition. Accordingly, 3 mM or higher 3-AT was selected as the screening pressure for subsequent droplet sorting to minimize background noise.

**Fig. 6:**
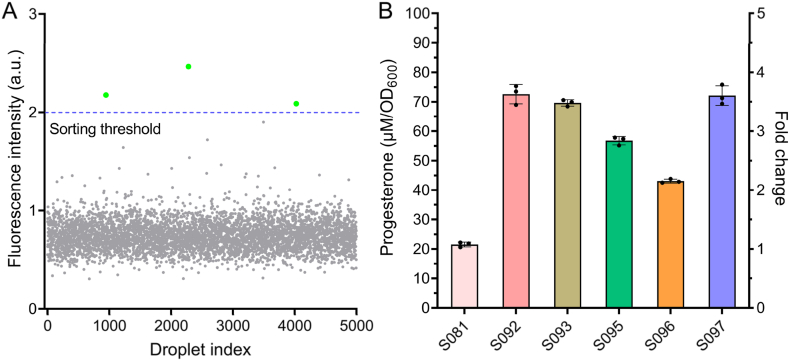
Screening of *S. cerevisiae* mutants using a FADS platform and validation by HPLC analysis. (A) Fluorescence distribution of 5000 droplets randomly sampled from a library of >1 × 10^6^ droplets. Most droplets exhibited baseline fluorescence (0.4–1.0 a.u.), while ∼0.06 % showed higher fluorescence (up to 2.47 a.u.). A threshold of 2.0 a.u. (blue dashed line) was applied to selectively recover high-fluorescence droplets. (B) Recovered clones were cultured in SD/–Ura medium supplemented with 4 % HP-β-CD, and progesterone production was assessed by HPLC using 1 mM pregnenolone as substrate. Mutants S092, S093, S095, S096, and S097 exhibited 2.00–3.37-fold higher biotransformation efficiency than the wild-type S081, with S092 producing the highest titer (72.59 μM/OD_600_).

Logarithmic-phase S081 cells were subjected to atmospheric and ARTP treatment for 0–240 s, achieving 95.06 % lethality at 90 s and 99.83 % at 120 s (Fig. S4). Ninety seconds was selected for three rounds of cumulative mutagenesis, balancing mutation efficiency and cell survival to generate sufficient genetic diversity. Fluorescence analysis of 5000 randomly sampled droplets from a population exceeding 1 × 10^6^ revealed a single baseline peak at 0.4–1.0 a.u., with ∼0.06 % reaching 2.47 a.u., representing potential high-producing variants (Fig. 6A). A selection threshold of 2.0 a.u. was applied, and microscopy confirmed that approximately 80 % of high-fluorescence droplets were successfully collected, demonstrating efficient sorting. Based on the proportion of target droplets before sorting (0.06 %) and the fraction of confirmed high-producing variants among the recovered droplets (∼10 %), the enrichment of high-producing variants was estimated to be ∼167-fold, highlighting the effectiveness of the microfluidic screening system.

HPLC quantification revealed that mutants S092, S093, S095, S096, and S097 exhibited 3.37-, 3.23-, 2.63-, 2.00-, and 3.35-fold increases in pregnenolone-to-progesterone conversion relative to wild-type S081 under validation conditions using 1 mM pregnenolone. Among these, S092 achieved the highest titer of 72.59 μM/OD_600_, exceeding the 900 μM substrate concentration used during droplet screening. This titer, obtained under low-copy CEN/ARS plasmid expression, reflects the relative efficiency of wild-type and mutant strains rather than representing industrial-level production. The high-producing mutants S092, S093, and S097 also maintained their enhanced progesterone production over seven consecutive generations, demonstrating stable phenotypes without significant decline (Fig. S5).

Validation of the growth-coupled biosensor under these screening conditions showed that S092 exhibited robust growth in droplets, whereas S081 grew minimally, confirming that cell proliferation within droplets reflects progesterone production and enables selective enrichment of high-producing mutants (Fig. S6).

qRT-PCR analysis showed that 3β-HSD transcription levels in S092, S093, S095, S096, and S097 increased 2.65-, 2.10-, 1.77-, 1.66-, and 2.23-fold relative to S081 (Fig. S7), indicating that host genome mutations enhanced enzyme expression and catalytic activity without altering the TEF1 promoter or coding sequence. RNA-seq analysis of the evolved mutant S092 revealed significant upregulation of genes associated with membrane transport and uptake (*HXT1*, *HXT2*, *PHO89*, *PTR2*, *FIT1*, *CIS3*, *TOH1*) [39,40] and redox processes (*FRM2*, *BDH1*, *ADH5*, *GDH3*, *ARO8*, *ARO9*) (Table S6) [41,42]. To validate the RNA-Seq results, a subset of genes—including upregulated (*TPO2*, *HXT1*, *SUL1*, *RRI1*, *LIF1*), non-differentially expressed (*ADE12*, *SUI2*), and downregulated genes (*THI2*, *CTR3*, *SSA3*, *PHM8*, *YEH1*)—was randomly selected for qRT-PCR, which confirmed consistent transcriptional trends in S092 (Fig. S8). Together, these results suggest that the improved pregnenolone-to-progesterone conversion in the evolved strains is likely due to a combination of increased 3β-HSD expression and host-level physiological adaptations induced by ARTP-mediated genome-wide mutations. Although the 3β-HSD coding sequence and its promoter remained unchanged, host genome mutations may indirectly enhance 3β-HSD transcription. This may occur through increased intracellular substrate availability via upregulated membrane transport genes and optimization of the NAD^+^/NADH balance via redox-associated genes. These coordinated physiological changes likely create a more favorable environment for 3β-HSD catalysis. Although the precise contribution of individual mutations remains to be elucidated, this plausible mechanistic link explains how genome-wide host adaptation contributes to the observed increase in metabolic conversion efficiency.

In addition to host genetic improvements, platform optimization further enhanced screening efficiency and product formation. HP-β-CD demonstrated excellent aqueous solubility and stability, effectively improving the bioavailability of pregnenolone and progesterone [28]. Application of 3-AT as a selective pressure substantially reduced background fluorescence, eliminating the need for pre-recording wild-type fluorescence and simplifying the screening workflow [17,43]. Despite low-copy plasmid expression and substrate limitations, the fold improvement demonstrates reliable enrichment of high-producing variants. Furthermore, by tuning selective pressures, such as increasing 3-AT concentration or reducing IPTG levels, the biosensor-FADS platform is adaptable for iterative strain evolution, enabling continuous enrichment of higher-producing strains and enhancing the efficiency of synthetic and systems biotechnology applications.

### 3.6 Overall comparison of biosensor performance

Key performance metrics of the growth-coupled progesterone-responsive biosensor are summarized in Table 1. Unlike conventional biosensors restricted to water-soluble substrates and single fluorescence outputs, this system integrates growth and fluorescence readouts to dynamically evaluate the intracellular conversion of pregnenolone to progesterone. The combination of 3-AT selection and the LacI–LacO module minimizes basal expression, while IPTG induction enables fine-tuned modulation of signal output. The biosensor maintained robust performance in microfluidic droplets, where fluorescence and growth responses accurately represented intracellular progesterone accumulation and associated metabolic activity. Overall, the biosensor demonstrates a wide dynamic range, high specificity, and dual-signal functionality that are fully compatible with ultra-high-throughput FADS screening. Its integration into the FADS workflow extends biosensor applicability to poorly soluble steroid substrates and provides a modular framework for pathway engineering, strain evolution, and system-level optimization of steroid biotransformation in yeast.

**Table 1:** Comparison of conventional biosensors and growth-coupled progesterone-responsive biosensor.

Performance Metric	Conventional Biosensors	Growth-Coupled Progesterone-Responsive Biosensor
Substrate specificity	Common metabolites or enzyme substrates [9,11]	Specifically responds to in vivo-produced progesterone; pregnenolone alone does not activate the sensorr (Fig. 4, Fig. 5; Fig. S3)
Signal modality	Fluorescence or enzymatic activity [9,44]	Integrated fluorescence and growth readouts, quantitatively reflecting cellular response to progesterone (Fig. 4, Fig. 5)
Background suppression	Low or none (leaky expression, background noise) [11,23]	Strong: 3-AT suppresses basal growth; LacI–operator system enables IPTG-tunable regulation for growth-coupled screening (Fig. 1, Fig. 4; Figs. S3 and S6)
Dynamic range	Narrow, promoter-dependent [9,44]	Broad: fluorescence linearly correlated with progesterone; growth dose-dependent; IPTG-tunable (Fig. 4, Fig. 5)
High-throughput compatibility	Microplate assays or conventional FADS, prone to false positives [9,12,14]	Droplet microfluidics with growth-coupled selection; 3-AT adjustable for stringent ultra-high-throughput screening (Fig. 1, Fig. 6; Fig. S3)
In-droplet validation	Rarely performed [34,38]	Demonstrated: 3-AT effectively suppresses wild-type growth in droplets (Figs. S3 and S6)
Response kinetics	Moderate (12–24 h to detectable signal) [9,44]	Differentiation detectable at 60 h; incubation period adjustable (Fig. 4, Fig. 5)

Conventional biosensors are usually developed in standard media for water-soluble substrates. The growth-coupled FADS platform uses 4 % HP-β-CD to improve solubility of poorly soluble steroids, such as pregnenolone and progesterone, enabling high-throughput strain screening.

## 4 Conclusions

In this study, we developed a growth-coupled progesterone-responsive biosensor integrated with a FADS platform for strain improvement of *S. cerevisiae*. Coupled with ARTP mutagenesis, this system significantly enhanced the conversion of pregnenolone to progesterone, demonstrating the potential of biosensor-guided screening for steroid biotransformation. The biosensor links a progesterone-responsive transcription factor to a dual-reporter module (EGFP and His3), coupling fluorescence to growth. Incorporation of the LacI–LacO regulatory system and IPTG induction enabled tunable responsiveness, while 3-AT effectively suppressed background signals, improving mutant discrimination in microdroplet screening. Integration with FADS allowed high-throughput, single-cell screening and rapid identification of improved mutants.

The platform operated robustly in HP-β-CD–containing media, confirming its suitability for poorly soluble steroid substrates. The resulting mutants provide valuable resources for studying regulatory mechanisms and pathway optimization in yeast. Overall, this work provides a practical and versatile strategy for biosensor-based screening and strain improvement, with potential applications in enzyme engineering and metabolic optimization. Moreover, this study highlights the “sensing–decision–response” closed-loop screening paradigm, which provides a robust and scalable framework for intelligent and automated engineering of microbial cell factories. By integrating real-time sensing, feedback decision-making, and response modulation, this platform can accelerate the production of non-model natural products and other bioactive compounds, supporting rational strain development, dynamic pathway optimization, and future enzyme evolution.

## CRediT authorship contribution statement

**Yucheng Hu:** Writing – original draft, Visualization, Validation, Methodology, Investigation, Formal analysis, Data curation, Conceptualization. **Jinde Chen:** Software, Investigation. **Shaofang Tian:** Validation, Formal analysis. **Yang Zhang:** Validation, Formal analysis. **Zhiqian Zhang:** Investigation, Formal analysis. **Ao Jiang:** Software, Formal analysis. **Yi-Rui Wu:** Writing – original draft, Supervision, Conceptualization. **Baoshun Zhang:** Writing – review & editing, Writing – original draft, Visualization, Supervision, Resources, Project administration, Funding acquisition, Data curation, Conceptualization.

## Declaration of interest statement

The authors declare the following financial interests/personal relationships which may be considered as potential competing interests: Jinde Chen, Yang Zhang, Zhiqian Zhang, Ao Jiang and Yi-Rui Wu are currently employed by Tidetron Bioworks Technology (Guangzhou) Co., Ltd., Guangzhou Qianxiang Bioworks Co., Ltd.

## References

[1] Zhang Y., Zhu X., Wang N., Liu X., Wang L., Ning K. (2025). Synergy of traditional practices and modern technology: advancing the understanding and applications of microbial resources and processes in fermented foods. Trends Food Sci Technol.

[2] Yan X., Liu X., Zhao C., Chen G.-Q. (2023). Applications of synthetic biology in medical and pharmaceutical fields. Signal Transduct Targeted Ther.

[3] Zhang J., Li F., Liu D., Liu Q., Song H. (2024). Engineering extracellular electron transfer pathways of electroactive microorganisms by synthetic biology for energy and chemicals production. Chem Soc Rev.

[4] Sarnaik A., Liu A., Nielsen D., Varman A.M. (2020). High-throughput screening for efficient microbial biotechnology. Curr Opin Biotechnol.

[5] Chae T.U., Choi S.Y., Kim J.W., Ko Y.-S., Lee S.Y. (2017). Recent advances in systems metabolic engineering tools and strategies. Curr Opin Biotechnol.

[6] Thomas N., Belanger D., Xu C., Lee H., Hirano K., Iwai K., Polic V. (2025). Engineering highly active nuclease enzymes with machine learning and high-throughput screening. Cell Syst.

[7] Li T., Dong H., Li J., Wang H., Pu C., Chen S., Yang Z., Ren X., Liu X., Jin Z., Zhang D. (2025). Extending dynamic and operational range of the biosensor responding to l-carnitine by directed evolution. Synth Syst Biotechnol.

[8] Xu A., Zhang X., Cao S., Zhou X., Yu Z., Qian X., Zhou J., Dong W., Jiang M. (2022). Transcription-associated fluorescence-activated droplet sorting for Di-rhamnolipid hyperproducers. ACS Synth Biol.

[9] Szczebara F.M., Chandelier C., Villeret C., Masurel A., Bourot S., Duport C., Blanchard S., Groisillier A., Testet E., Costaglioli P. (2003). Total biosynthesis of hydrocortisone from a simple carbon source in yeast. Nat Biotechnol.

[10] Wang Y., Zhang R., Yao M., Xiao W., Wang Y., Yuan Y.-J. (2025). Transcriptomic studies on the product stress response revealed that YCF1 is a beneficial factor for progesterone production in *Yarrowia lipolytica*. Synth Syst Biotechnol.

[11] Qin L., He S., Hou J., Li G., Feng Y., Zhao M., Huang M. (2025). Adaptive laboratory evolution induces cell wall alterations for succinic acid tolerance in *Saccharomyces cerevisiae*. Bioresour Technol.

[12] Cleaver A., Luo R., Smith O.B., Murphy L., Schwessinger B., Brock J. (2025). High-throughput optimisation of protein secretion in yeast via an engineered biosensor. Trends Biotechnol.

[13] Reyes L.H., Gomez J.M., Kao K.C. (2014). Improving carotenoids production in yeast via adaptive laboratory evolution. Metab Eng.

[14] Cho S., Kang D.-K., Sim S., Geier F., Kim J.-Y., Niu X., Edel J.B., Chang S.-I., Wootton R.C.R., Elvira K.S., deMello A.J. (2013). Droplet-based microfluidic platform for high-throughput, multi-parameter screening of photosensitizer activity. Anal Chem.

[15] Li L., Zhang Q., Shi R., Yao M., Tian K., Lu F., Qin H.-M. (2024). Multidimensional combinatorial screening for high-level production of erythritol in *Yarrowia lipolytica*. Bioresour Technol.

[16] Chen C., Liu J., Yao G., Bao S., Wan X., Wang F., Wang K., Song T., Han P., Liu T., Jiang H. (2023). A novel, genetically encoded whole-cell biosensor for directed evolution of myrcene synthase in *Escherichia coli*. Biosens Bioelectron.

[17] Stella R.G., Gertzen C.G., Smits S.H., Gätgens C., Polen T., Noack S., Frunzke J. (2021). Biosensor-based growth-coupling and spatial separation as an evolution strategy to improve small molecule production of *Corynebacterium glutamicum*. Metab Eng.

[18] Wang R., Cress B.F., Yang Z., Hordines J.C., Zhao S., Jung G.Y., Wang Z., Koffas M.A.G. (2019). Design and characterization of biosensors for the screening of modular assembled naringenin biosynthetic library in *Saccharomyces cerevisiae*. ACS Synth Biol.

[19] Zhang K., Li M., Wang J., Huang G., Ma K., Peng J., Lin H., Zhang C., Wang H., Zhan T., Sun Z., Zhang X. (2024). Optimizing enzyme properties to enhance dihydroxyacetone production via methylglyoxal biosensor development. Microb Cell Fact.

[20] Wang G., Øzmerih S., Guerreiro R., Meireles A.C., Carolas A., Milne N., Jensen M.K., Ferreira B.S., Borodina I. (2020). Improvement of cis,cis-muconic acid production in *Saccharomyces cerevisiae* through biosensor-aided genome engineering. ACS Synth Biol.

[21] Grazon C., Baer R.C., Kuzmanović U., Nguyen T., Chen M., Zamani M., Chern M., Aquino P., Zhang X., Lecommandoux S., Fan A., Cabodi M., Klapperich C., Grinstaff M.W., Dennis A.M., Galagan J.E. (2020). A progesterone biosensor derived from microbial screening. Nat Commun.

[22] Liu K., Zhang Y., Liu K., Zhao Y., Gao B., Tao X., Zhao M., Wang F.-Q., Wei D. (2022). De novo design of a transcription factor for a progesterone biosensor. Biosens Bioelectron.

[23] Ottoz D.S.M., Rudolf F., Stelling J. (2014). Inducible, tightly regulated and growth condition-independent transcription factor in *Saccharomyces cerevisiae*. Nucleic Acids Res.

[24] Michael Lee K., DaSilva N.A. (2005). Evaluation of the *Saccharomyces cerevisiae* ADH2 promoter for protein synthesis. Yeast.

[25] Gietz R.D., Schiestl R.H. (2007). High-efficiency yeast transformation using the LiAc/SS carrier DNA/PEG method. Nat Protoc.

[26] Mazumder M., McMillen D.R. (2014). Design and characterization of a dual-mode promoter with activation and repression capability for tuning gene expression in yeast. Nucleic Acids Res.

[27] Zhang Q., Wang T., Zhou Q., Zhang P., Gong Y., Gou H., Xu J., Ma B. (2017). Development of a facile droplet-based single-cell isolation platform for cultivation and genomic analysis in microorganisms. Sci Rep.

[28] Fenyvesi É., Puskás I., Szente L. (2019). Applications of steroid drugs entrapped in cyclodextrins. Environ Chem Lett.

[29] Souza-Moreira T.M., Navarrete C., Chen X., Zanelli C.F., Valentini S.R., Furlan M., Nielsen J., Krivoruchko A. (2018). Screening of 2A peptides for polycistronic gene expression in yeast. FEMS Yeast Res.

[30] Žunar B., Mosrin C., Bénédetti H., Vallée B. (2022). Re-engineering of CUP1 promoter and Cup2/Ace1 transactivator to convert *Saccharomyces cerevisiae* into a whole-cell eukaryotic biosensor capable of detecting 10 nM of bioavailable copper. Biosens Bioelectron.

[31] Titz B., Thomas S., Rajagopala S.V., Chiba T., Ito T., Uetz P. (2006). Transcriptional activators in yeast. Nucleic Acids Res.

[32] Furuyama K., Sassa S. (2000). Interaction between succinyl CoA synthetase and the heme-biosynthetic enzyme ALAS-E is disrupted in sideroblastic anemia. J Clin Investig.

[33] Stekovic S., Ruckenstuhl C., Royer P., Winkler-Hermaden C., Carmona-Gutierrez D., Fröhlich K.U., Kroemer G., Madeo F. (2017). The neuroprotective steroid progesterone promotes mitochondrial uncoupling, reduces cytosolic calcium and augments stress resistance in yeast cells. Microb Cell.

[34] Guo L., Zeng W., Xu S., Zhou J. (2020). Fluorescence-activated droplet sorting for enhanced pyruvic acid accumulation by *Candida glabrata*. Bioresour Technol.

[35] Jiang J., Yang G., Ma F. (2023). Fluorescence coupling strategies in fluorescence-activated droplet sorting (FADS) for ultrahigh-throughput screening of enzymes, metabolites, and antibodies. Biotechnol Adv.

[36] Li H., Zhang W., Han Y., Tang G., Lu F., Qin H.-M. (2025). Programming a bacterial biosensor for directed evolution of tryptophan hydroxylase via high-throughput droplet sorting. Biosens Bioelectron.

[37] Bowman E.K., Wagner J.M., Yuan S.-F., Deaner M., Palmer C.M., D’Oelsnitz S., Cordova L., Li X., Craig F.F., Alper H.S. (2021). Sorting for secreted molecule production using a biosensor-in-microdroplet approach. Proc Natl Acad Sci USA.

[38] Liu H., Xu X., Peng K., Zhang Y., Jiang L., Williams T.C., Paulsen I.T., Piper J.A., Li M. (2021). Microdroplet enabled cultivation of single yeast cells correlates with bulk growth and reveals subpopulation phenomena. Biotechnol Bioeng.

[39] Rossi G., Sauer M., Porro D., Branduardi P. (2010). Effect of HXT 1 and HXT 7 hexose transporter overexpression on wild-type and lactic acid producing *Saccharomyces cerevisiae* cells. Microb Cell Fact.

[40] Özcan S., Johnston M. (1999). Function and regulation of yeast hexose transporters. Microbiol Mol Biol Rev.

[41] Hou J., Lages N.F., Oldiges M., Vemuri G.N. (2009). Metabolic impact of redox cofactor perturbations in *Saccharomyces cerevisiae*. Metab Eng.

[42] Heux S., Cachon R., Dequin S. (2006). Cofactor engineering in *Saccharomyces cerevisiae*: expression of a H2O-forming NADH oxidase and impact on redox metabolism. Metab Eng.

[43] Jain A., Stavrakis S., deMello A. (2024). Droplet-based microfluidics and enzyme evolution. Curr Opin Biotechnol.

[44] Song Y., Lin B., Tian T., Xu X., Wang W., Ruan Q., Guo J., Zhu Z., Yang C. (2019). Recent progress in microfluidics-based biosensing. Anal Chem.

