# Supplementary material for: A growth-coupled progesterone-responsive biosensor for high-throughput microfluidic screening in Saccharomyces cerevisiae
Source: Synth Syst Biotechnol. 2026 Jan 2;12:301–11. doi: 10.1016/j.synbio.2025.12.004 (PMC12805089; doi:10.1016/j.synbio.2025.12.004)
Supplement: Multimedia component 1 [file mmc1.docx]

Supplementary information

**A growth-coupled progesterone-responsive biosensor for high-throughput microfluidic screening in *Saccharomyces cerevisiae***

Yucheng Hu^a^, Jinde Chen^b^, Shaofang Tian^a^, Yang Zhang^b^, Zhiqian Zhang^b^, Ao Jiang^b^, Yi-Rui Wu^b,*^, Baoshun Zhang^a,*^

^a^*College of Pharmaceutical Sciences, Southwest University, Chongqing 400715, China*

^b^*Tidetron Bioworks Technology (Guangzhou) Co., Ltd., Guangzhou Qianxiang Bioworks Co., Ltd., Guangzhou 510000, China*

*Corresponding authors.

E-mail addresses: zbs360@swu.edu.cn (Baoshun Zhang), oxwyr1981@gmail.com (Yi-Rui Wu).


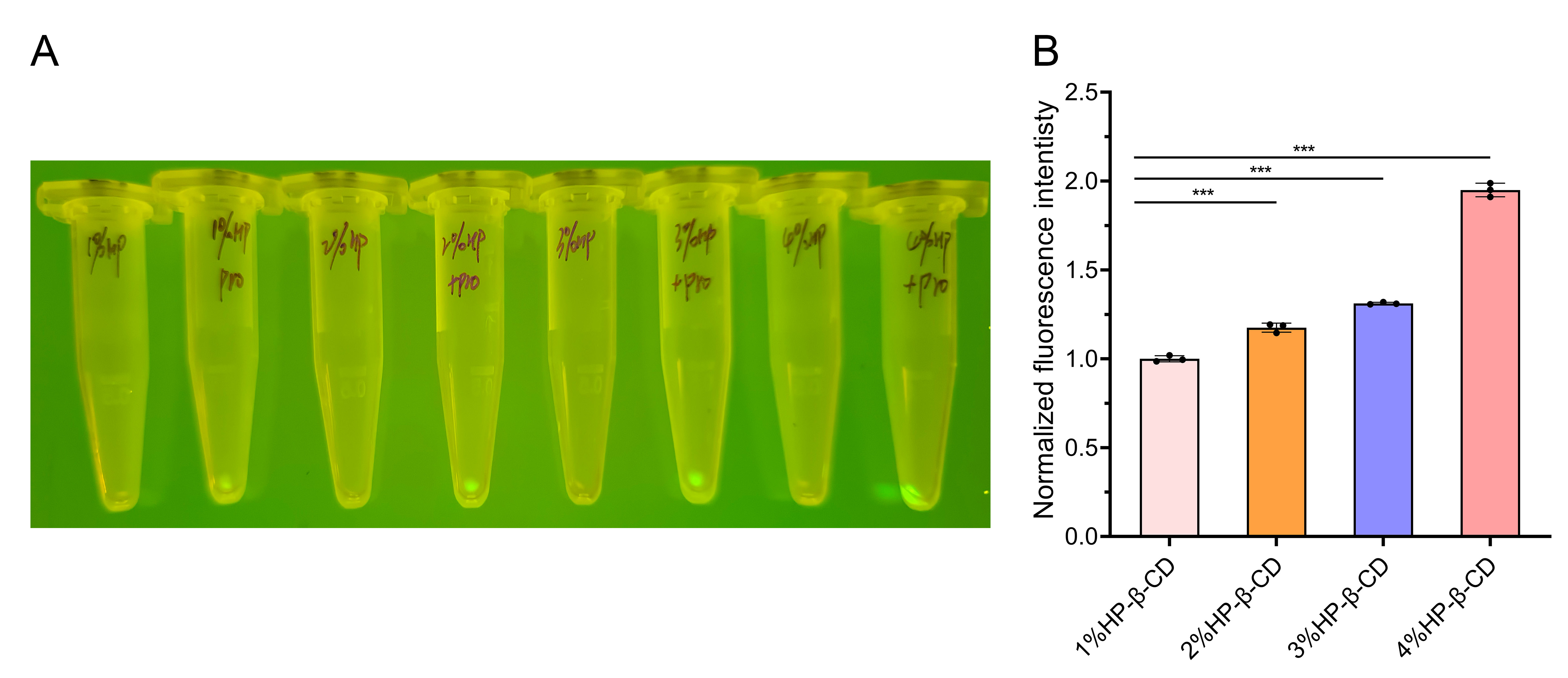


**Fig. S1.** Effect of HP-β-CD supplementation on fluorescence intensity of strain S077.

(A) Fluorescence images of strain S077 cultured in SD/–Leu medium supplemented with 1%, 2%, 3%, or 4% HP-β-CD, with or without 0.2 mM progesterone, under blue light excitation (λ = 488 nm). (B) Quantification of fluorescence intensities normalized to the 1% HP-β-CD condition. Data represent mean ± SD (n = 3). Statistical significance compared to 1% HP-β-CD was determined by one-way ANOVA followed by Tukey’s multiple comparisons test. Significance levels are denoted as **p* < 0.05, ***p* < 0.01 and ****p* < 0.001.

**
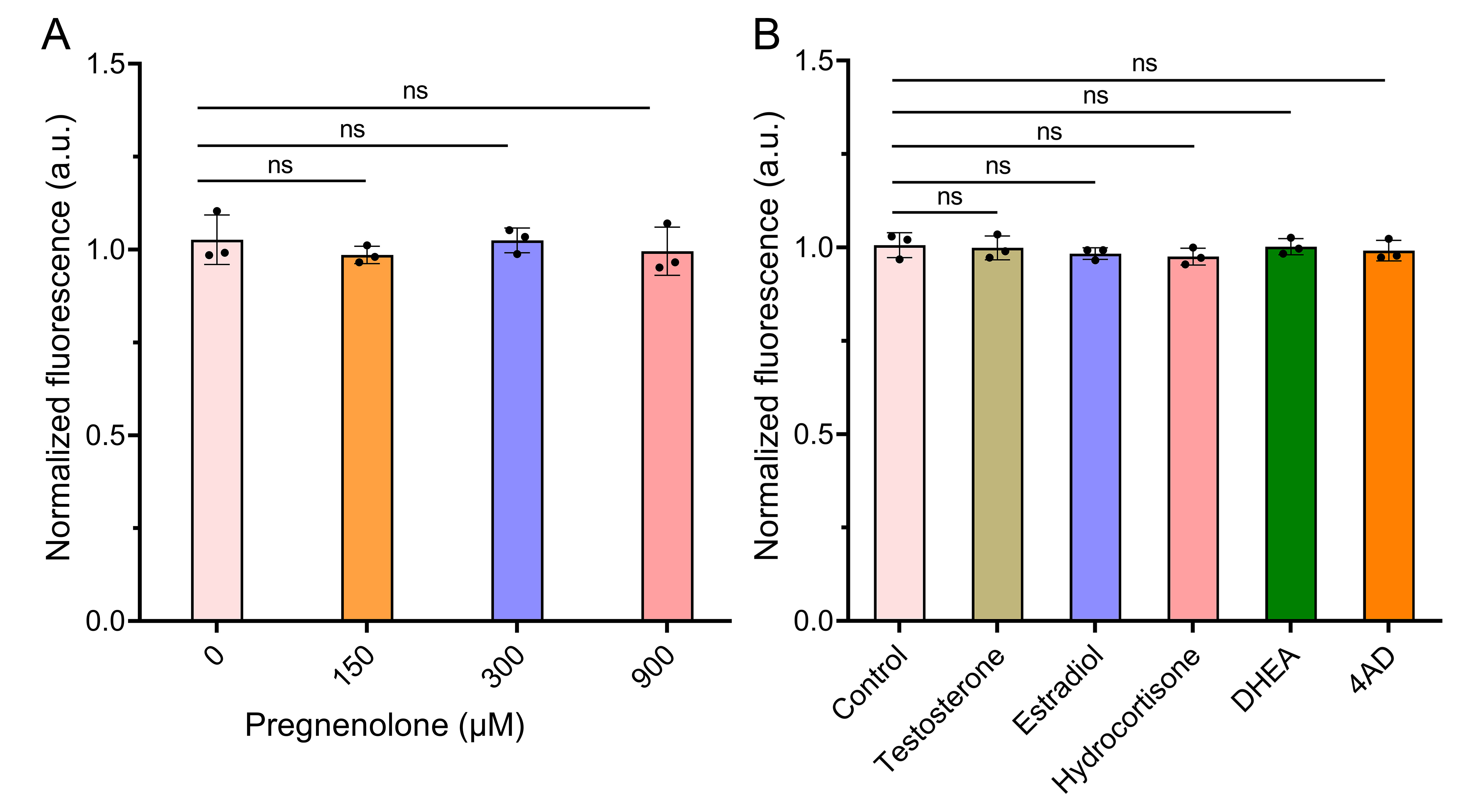
**

**Fig. S2.** Specificity evaluation of the growth-coupled progesterone biosensor in S077.

(A) Fluorescence response of strain S077 treated with pregnenolone at final concentrations of 0, 150, 300, or 900 μM. Fluorescence intensities were normalized to the no-compound control for each concentration, and no significant changes were observed, indicating that pregnenolone did not significantly activate the biosensor within the tested concentration range. (B) Fluorescence response of strain S077 treated with structurally related steroid compounds, including testosterone, estradiol, hydrocortisone, dehydroepiandrosterone (DHEA), and androstenedione (4-AD), each at a final concentration of 100 μM. Fluorescence intensities were normalized to the no-compound control for each compound, and no significant changes were observed, indicating that these compounds did not significantly activate the biosensor within the tested concentration. S077 cells were cultured in SD medium lacking leucine and supplemented with 4% HP-β-CD. Pregnenolone was added for panel A, and the structurally related steroid compounds were added for panel B. After 16 h of incubation, fluorescence was measured, normalized to the respective no-compound control, and analyzed by one-way ANOVA. Data represent mean ± SD (n = 3). Statistical significance for all comparisons is reported as *p* > 0.05.


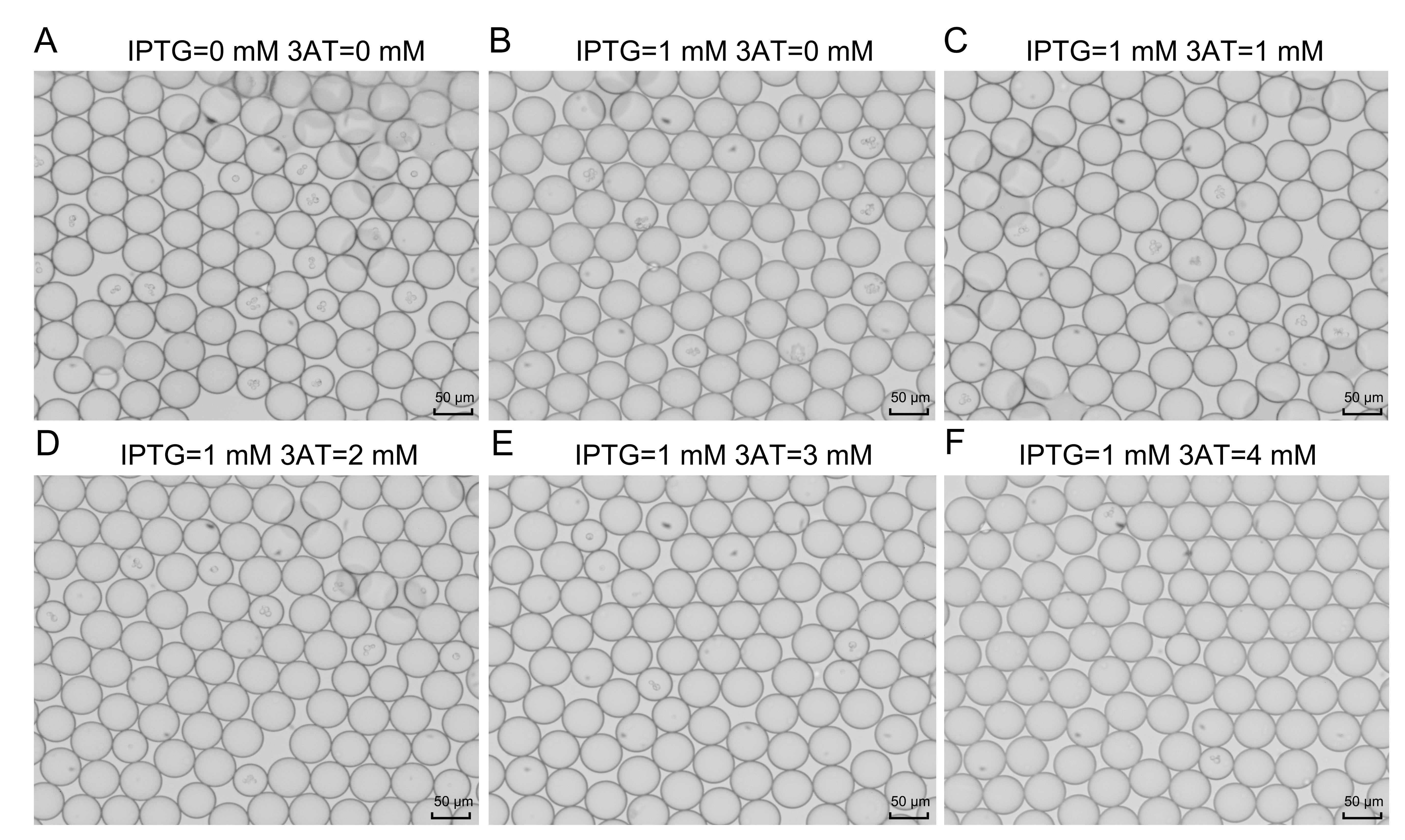


**Fig. S3.** Optimization of sorting conditions for a growth-coupled progesterone biosensor in strain S081 via microfluidic droplets. (A) IPTG = 0 mM, 3-AT = 0 mM. (B–F) IPTG = 1 mM, with 3-AT concentrations of 0, 1, 2, 3, and 4 mM, respectively. All experiments were performed in SD medium supplemented with 4% HP-β-CD and lacking Leu, Ura, and His, with 900 μM pregnenolone as the final substrate concentration. Fig. S3. Optimization of microfluidic droplet sorting conditions for the growth-coupled progesterone biosensor in S081.


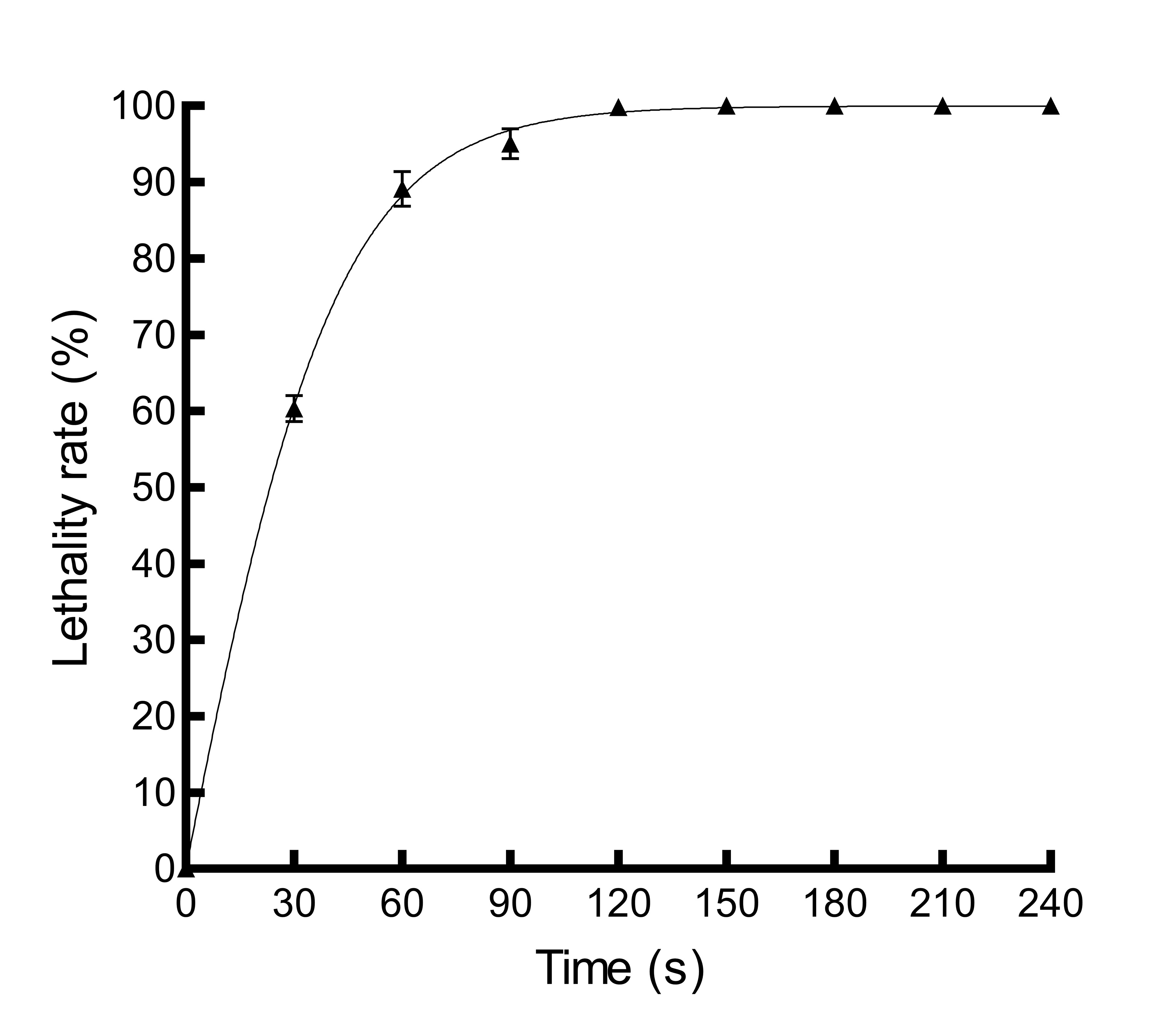


Fig. S4. Optimization of ARTP-induced lethality in strain S081. ARTP treatment was performed at a power setting of 120 W and an airflow rate of 10 SLM.

**
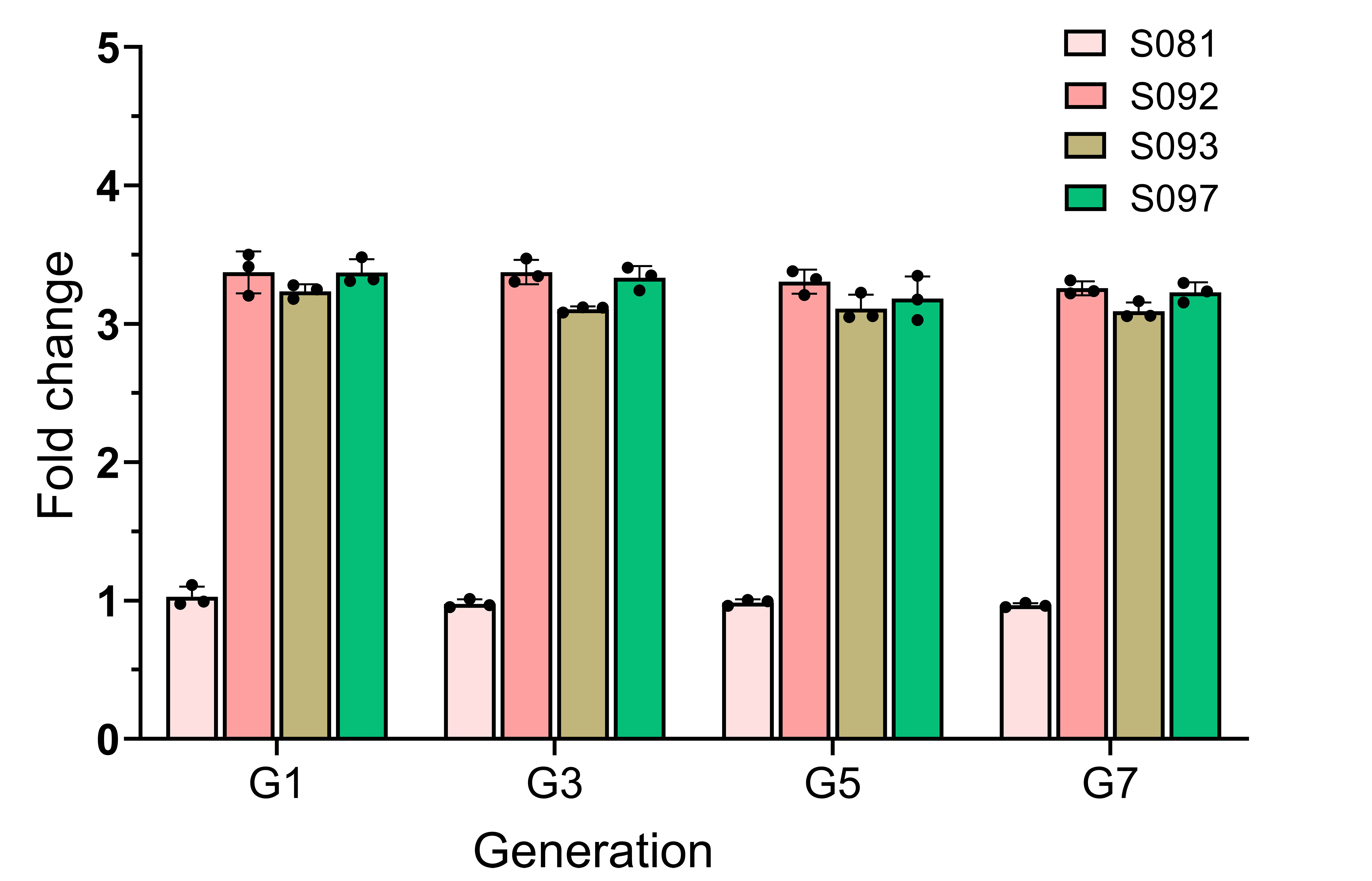
**

**Fig. S5.** Long-term genetic stability of progesterone-overproducing mutants.

Fold-change values of progesterone production for mutants S092, S093, and S097 across seven consecutive generations. Strains were serially passaged in SD/–Ura medium (1% inoculum, 30 °C, 250 rpm, 24 h per generation) and measured in SD medium supplemented with 4% HP-β-CD and 1 mM pregnenolone. Values were normalized to S081 at G1 (= 1). Data are presented as mean ± SD from three independent biological replicates (n = 3). Fold-changes of all subsequent generations relative to the first generation were within 5%, demonstrating stable phenotypes.


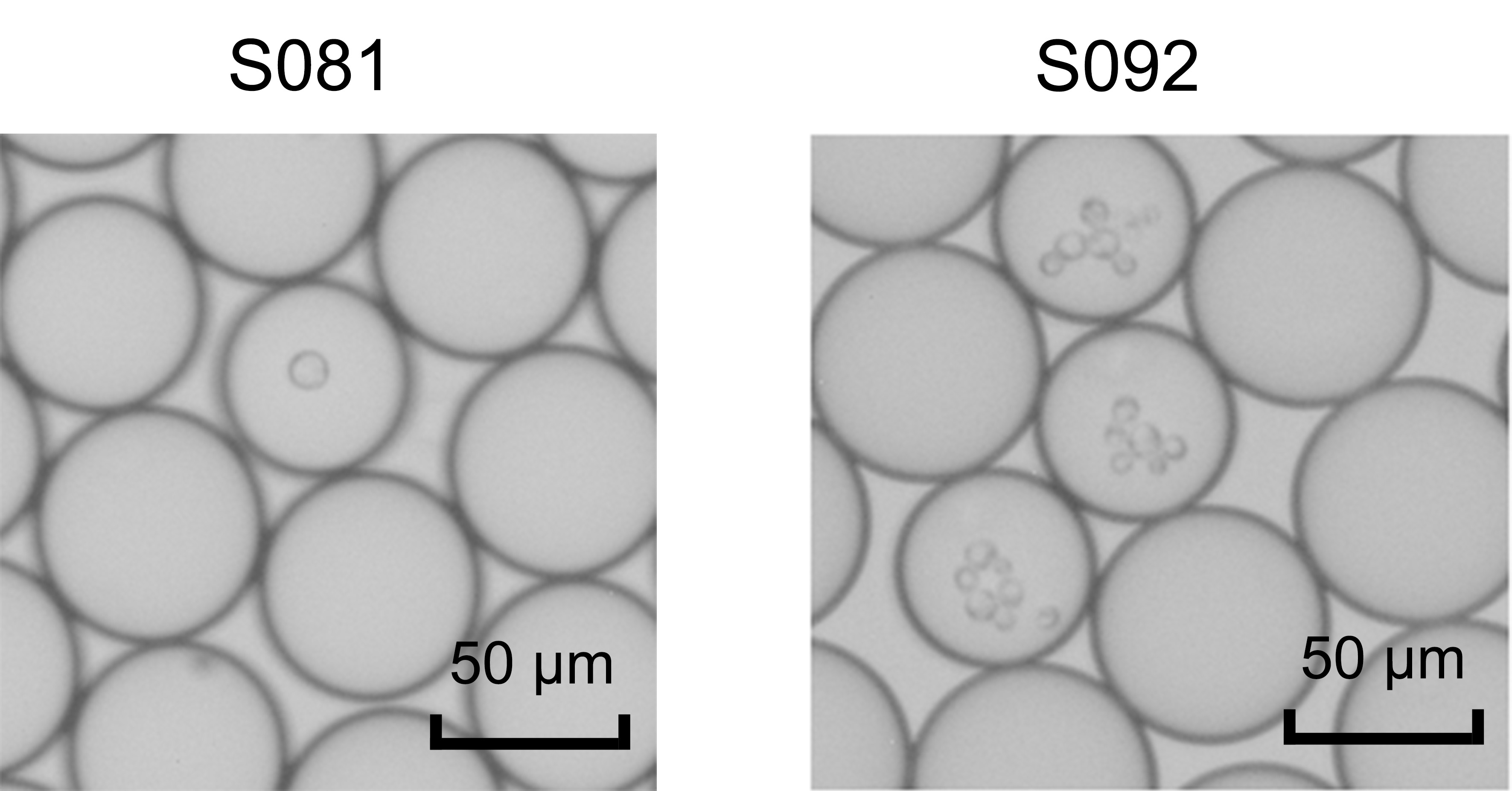


**Fig. S6.** Validation of growth-coupled biosensor via droplet growth.

S092 (high-producing mutant) and wild-type S081 were separately encapsulated in microfluidic droplets containing SD medium with 4% HP-β-CD, lacking leucine, uracil, and histidine, supplemented with 900 μM pregnenolone, 1 mM IPTG, and 3 mM 3-AT. After incubation at 30 °C for 60 h, droplets with S092 showed robust growth, whereas droplets with S081 exhibited minimal growth, confirming that cell proliferation within droplets reflects progesterone production and enables selective enrichment of high-producing mutants. Scale bar, 50 μm.


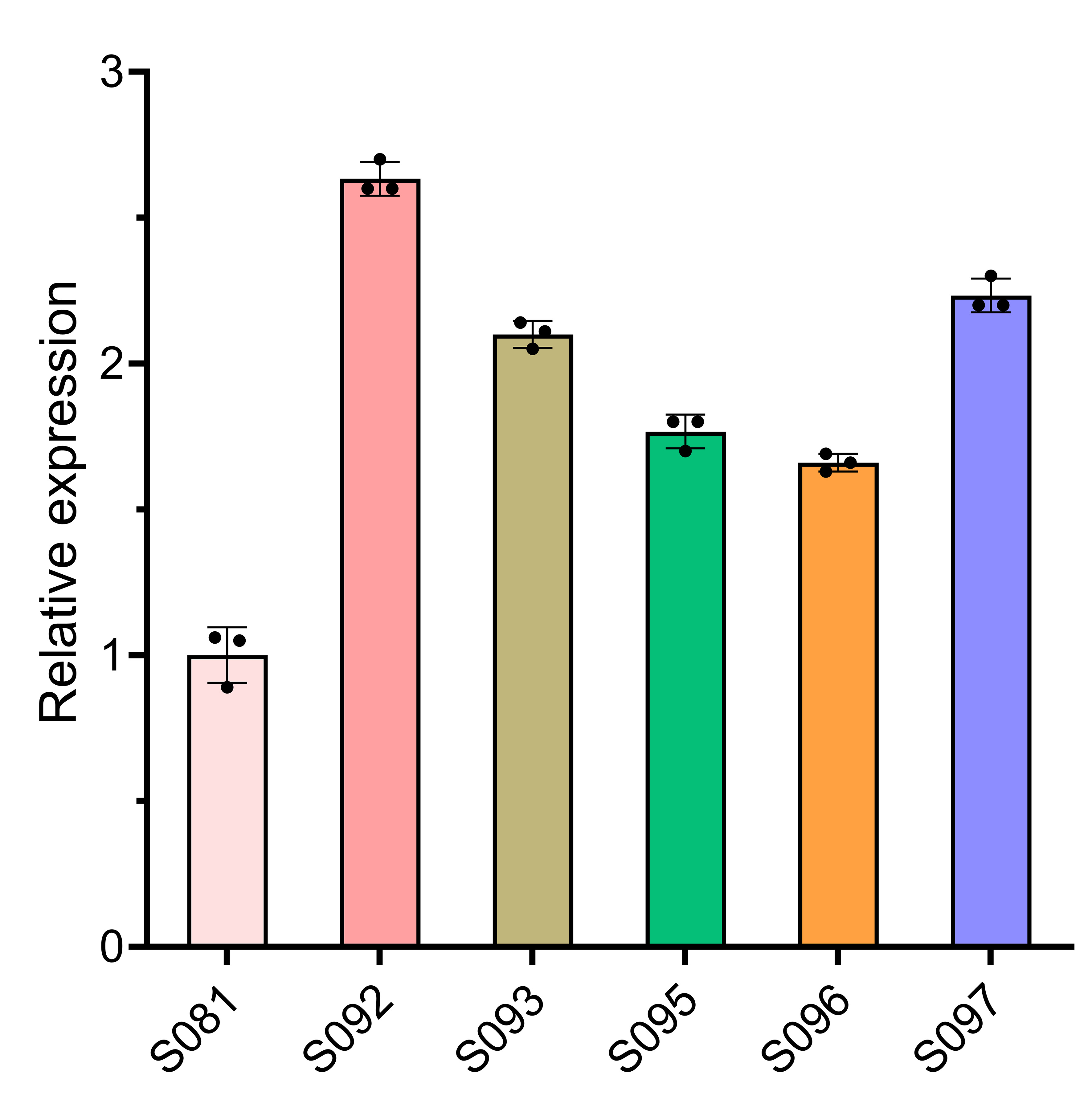


**Fig. S7.** Transcriptional analysis of *3β-HSD* in wild-type and mutant *S. cerevisiae* strains. Relative mRNA expression levels of *3β-HSD* were determined in wild-type strain S081 and mutant strains S092, S093, S095, S096, and S097 using qRT-PCR. *ALG9* was employed as the internal reference gene due to its stable expression across all strains under the experimental conditions. The expression level of 3β-HSD in S081 was normalized to 1. Compared with S081, the transcription levels of *3β-HSD* in S092, S093, S095, S096, and S097 increased by 2.65-, 2.10-, 1.77-, 1.66-, and 2.23-fold, respectively. Total RNA extraction, cDNA synthesis, and qRT-PCR were performed following standard procedures. Data are presented as mean ± SD from three independent biological replicates (n = 3), and relative expression levels were calculated using the 2^-ΔΔCt^ method.

**
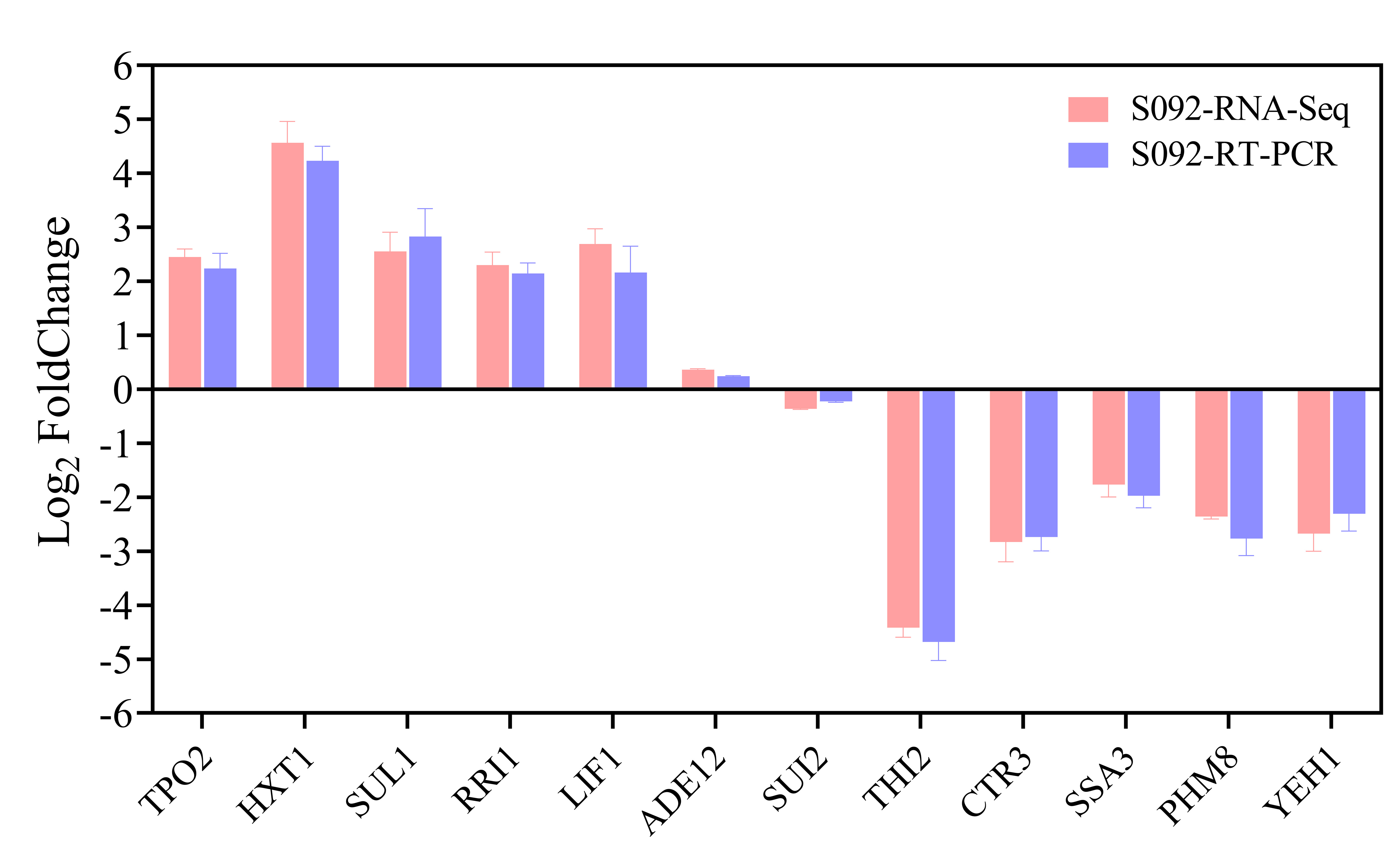
**

**Fig. S8.** Validation of RNA-Seq transcriptional trends by qRT-PCR in S092.

A subset of genes was randomly selected for qRT-PCR validation, including upregulated genes (*TPO2*, *HXT1*, *SUL1*, *RRI1*, *LIF1*), non-differentially expressed genes (*ADE12*, *SUI2*), and downregulated genes (*THI2*, *CTR3*, *SSA3*, *PHM8*, *YEH1*). Relative mRNA expression levels were normalized to the internal reference gene ALG9. qRT-PCR results confirmed that transcriptional trends in S092 were consistent with RNA-Seq data. Data are presented as mean ± SD from three independent biological replicates, and relative expression levels were calculated using the 2^-ΔΔCt^ method.

**Table S1.**

Plasmids used in this study.

| Plasmids | Description | Selection Marker | Source |
| --- | --- | --- | --- |
| pML107 | N/A | *His3, AmpR* | Addgene#67639 |
| pCas | N/A | *KanMX, KanR* | Addgene#60847 |
| 58430 | *FRP467-P_ACT1(-1-520)_-LexA-ER-haVP16* | *His3, AmpR* | Addgene#58430 |
| 58434 | *FRP793_insul-(lexA-box)_4_-P_minCYC1_-Citrine-T_CYC1_* | *URA3*, *AmpR* | Addgene#58434 |
| 130873 | *REV1p-TP-DNAP1* | *His3, KanR* | Addgene#130873 |
| pUC19 | N/A | N/A, *AmpR* | Lab store |
| pML107T | N/A | *Trp1*, *AmpR* | Lab store |
| pYES2 | N/A | *URA3*, *AmpR* | Lab store |
| HY-E010 | *pRNR2-trp1-sgRNA* | *Leu2*, *AmpR* | This work |
| HY-E011 | *pRNR2-ayr1-sgRNA* | *Leu2*, *AmpR* | This work |
| HY-E012 | *pRNR2-ura3-sgRNA* | *Leu2*, *AmpR* | This work |
| HY-e013 | *pRNR2-atf2-sgRNA* | *Trp1*, *AmpR* | This work |
| HY-E014 | *pRNR2-his3-sgRNA* | *Trp1*, *AmpR* | This work |
| HY-E034 | *yCE-P_TEF1_-3β-HSD-T_ADH1_* | *HIS3*, *KanR* | This work |
| HY-E089 | *FRP793_insul-(LexA-box)_4_-P_minCYC1_-eGFP-T_CYC1_* | *Leu2*, *AmpR* | Addgene#58434 |
| HY-E090 | *FRP793_insul-(LexA-box)_4_-P_minCYC1_-his3-2a-eGFP-T_CYC1_* | *Leu2*, *AmpR* | This work |
| HY-E107 | *pRNR2-ura3-sgRNA* | *Trp1*, *AmpR* | This work |
| HY-E125 | *pUC19-ura3-P_ACT1_-LexA-pro-Vp16* | *N/A, AmpR* | This work |
| HY-E163 | *yCE-P_TEF1_-3β-HSD-T_ADH1_* | *URA3*, *KanR* | This work |
| HY-E164 | *FRP793_insul-P_CUP1_-LacI-(lexA)_4_-box-LacO-P_minCYC1_-his3-2A-eGFP-T_CYC1_* | *Leu2*, *AmpR* | This work |
| HY-E165 | *FRP793_insul-P_CUP1_-LacI-(lexA)_4_-box-(LacO)_2_-P_minCYC1_-his3-2A-eGFP-T_CYC1_* | *Leu2*, *AmpR* | This work |
| HY-E166 | *FRP793_insul-P_CUP1_-LacI-(lexA)_4_-box-(LacO)_3_-P_minCYC1_-his3-2A-eGFP-T_CYC1_* | *Leu2*, *AmpR* | This work |

Table S2.

| Strain | Genotype | Parent Strain | Source |
| --- | --- | --- | --- |
| CENPK2-1c | MATa *ura3-52 trp1-289 leu2-3,112 his3∆1,* MAL2-8c*,* SUC2*,* ρ^+^ | N/A | Lab store |
| BY4741 | MATa *his3∆1 leu2∆0 met15∆0 ura3∆0,* ρ+ | N/A | Coolaber |
| S059 | MATa *his3∆1 leu2-3,112 trp1-289 ura3-52 ayr1∆* | CENPK2-1c | This work |
| S060 | MATa *his3∆1 leu2-3,112 trp1-289 ura3-52 ayr1∆ atf2∆* | S059 | This work |
| S061 | MATa *his3∆0 leu2-3,112 trp1-289 ura3-52 ayr1∆ atf2∆* | S060 | This work |
| S062 | MATa *his3∆0 leu2-3,112 trp1-289 ura3∆0 ayr1∆ atf2∆* | S061 | This work |
| S063 | MATa *his3∆0 leu2-3,112 trp1∆0 ura3∆0 ayr1∆ atf2∆* | S062 | This work |
| S064 | MATa *his3∆0 leu2-3,112 trp1∆0 ayr1∆ atf2∆* | S063 | This work |
| S065 | S064, (*ura3∆*::*P_ACT1_-LexA-pro-vp16*) | S064 | This work |
| S067 | S065, (KI.*LEU2* :: HY-E089) | S065 | This work |
| S068 | S065, (KI.*LEU2*:: HY-E090) | S065 | This work |
| S077 | S065, (KI.*LEU2*:: HY-E164) | S065 | This work |
| S078 | S065, ( KI.*LEU2*:: HY-E165) | S065 | This work |
| S079 | S065, (KI.*LEU2*:: HY-E166) | S065 | This work |
| S081 | S077, carrying HY-E163 plasmid (3β-HSD) | S077 | This work |
| S092 | ARTP mutant, carrying HY-E163 plasmid (3β-HSD) | S081 | This work |
| S093 | ARTP mutant, carrying HY-E163 plasmid (3β-HSD) | S081 | This work |
| S095 | ARTP mutant, carrying HY-E163 plasmid (3β-HSD) | S081 | This work |
| S096 | ARTP mutant, carrying HY-E163 plasmid (3β-HSD) | S081 | This work |
| S097 | ARTP mutant, carrying HY-E163 plasmid (3β-HSD) | S081 | This work |

Engineered *Saccharomyces cerevisiae* strains used in this study.

Table S3.

Primers used in this study.

| Primer name | Sequences (5' → 3') |
| --- | --- |
| 25-F | TGAGCAGGCAAGATAAACGAGTTTTAGAGCTAGAAATAGC |
| 26-R | TCGTTTATCTTGCCTGCTCAGATCATTTATCTTTCACTGC |
| 27-F | TTAGCTTCTCGACGTGGGCC |
| 28-R | TATAGAATGAAGGAGGCCAAGAGTAATAGA |
| 29-F | TTGGCCTCCTTCATTCTATACGTGTCATTC |
| 30-R | GTCCAATGGAGGTGAGGTAAATT |
| 31-F | TCTTTCCTGCTTTGAATTAGTCG |
| 32-R | TGTGAAGCTGAACCGTTGATTTTTACTGAT |
| 33-F | ATCAACGGTTCAGCTTCACAGAAACCTCAT |
| 34-R | TCAAAAGGCCTGCAGGCAAGTGCA |
| 46-F | TTTTGTAGTGCCCTCTTGGGCTAGCGGTAAAGGTGCGCAT |
| 47-R | ATTGACCACACCTCTACCGGCATGCCGAGCAAATGCCTGC |
| 52-F | TTTGCATTATTTGAAACTAGGGAA |
| 53-R | ATTTTGTTTGGATTTGGTTAGATT |
| 54-F | CCCTTGTTTGATTCAGAAGCGTTTTAGAGCTAGAAATAGC |
| 55-R | GCTTCTGAATCAAACAAGGGGATCATTTATCTTTCACTGC |
| 57-F | GTTGAAGTTGGCTGGTGACGTTGAACTTAACCCAGGTCCAATGTCTAAAGGTGAAGAATTATTCACTGG |
| 93-sg-F | AGTCAAATTGCAGTACTCTGGTTTTAGAGCTAGAAATAGC |
| 94-sg-R | CAGAGTACTGCAATTTGACTGATCATTTATCTTTCACTGC |
| 115-F | TATCAAGCTTATGACAGAGCAGAAAGCCCTAGTA |
| 118-R | GTCACCAGCCAACTTCAACAAAGAGAAGTTAGTAGCACCCATAAGAACACCTTTGGTGGA |
| 116-R | GCTCTGTCATAAGCTTGATATCGAATTCCTGCAGCCCG |
| 255-F | GTGGAATTGTGAGCGGATAACAACTTTTCTCTTCTTTCCTTATACATT |
| 256-R | TTATCCGCTCACAATTCCACACAAAGAAAACAAGAGTTTTATATACAT |
| 267-R | CCCGCTTCTCTAGAGCGGCCGCCGAATCAAAAAAATTTCAA |
| 268-F | TACGGTGGGAAGCTTTAAGCGGCCGCATACTAAACTCACAAATTA |
| 269-F | TCTAGAGGATCCCCGGGTACCAATCGATCAAGACAGACAGC |
| 270-R | ACTGAGAGTGCACCATATGGGTACCTACAGAAGGATCCCCACCTA |
| 282-F | GTCTGCTTAGGCCTACTAGTGAGCGACCTCATGCTATA |
| 283-R | ACTAGTAGGCCTAAGCAGACGCTACTAAGGAAAACAA |
| 302-F | GGGAAGGCCTCAGCTTCGGCCGTGTACCCAGCTTTTGTTC |
| 304-F | CAGGGAGCTCCCACACACCATAGCTTCAAAAT |
| 305-R | TGGTGTGTGGGAGCTCCCTGATGCGGTATTTTCTCCTT |
| 401-R | CAATCAATCAATGAAACCAGTAACATTGTATGATGTCGCAGA |
| 402-F | CTGGTTTCATTGATTGATTGATTGATTGTAC |
| BDH1-yz-for | GAACAGGGAAGCCGATAATGAG |
| BDH1-yz-rev | CCAGTTTGTCTTCCAGAGCC |
| Leu2insert-F | AAGCCCGTCATCGAGGAGAACTTCTAGTATAT |
| Leu2TInsert-R | ACCGCATATGTTTTTAAGCAAGGATTTTCTTA |
| GFP-F | TATCAAGCTTATGTCTAAAGGTGAAGAATT |
| GFPZ-R | CTGTCTCGAGTTATTTGTACAATTCATCCAT |
| Leu2Tinsert-F | TGCTTAAAAACATATGCGGTGTGAAATACCGCACAGAT |
| YEGFP-R | CTTTAGACATAAGCTTGATATCGAATTCCTGCAG |
| GFPZ-F | GTACAAATAACTCGAGACAGGCCCCTTTTCCTTT |
| Leu2insertt-R | TTCTCCTCGATGACGGGCTTGTCTGCTCCCGGCATCCG |
| Ura3-sg-F | CCTTCGTTCTTCCTTCTGCTGTTTTAGAGCTAGAAATAGCAA |
| Ura3-sg-R | AGCAGAAGGAAGAACGAAGGGATCATTTATCTTTCACTGCGG |
| ACT1-F | GGCCGCTCTAGAGAAGCGGGTAAG |
| VP16AD-R | TTAAAGCTTCCCACCGTA |
| PUC19ZH-F | CCATATGGTGCACTCTCAGT |
| PUC19ZH-R | GTACCCGGGGATCCTCTAGAGTCG |
| AYR1P-F | TACAGCCATATAAACACGT |
| AYR1D-R | TGGTGAAACAACTCTTTGAAATCCTGATCA |
| AYR1D-F | TTCAAAGAGTTGTTTCACCATCGTCAATAG |
| AYR1T-R | CTTGCTATAGTGTCTTATTC |
| AYR1-SG-R | TCAGGGAAGTTGTATATTGAGATCATTTATCTTTCACTGC |
| AYR1-R | TCAGGGAAGTTGTATATTGAGATCATTTATCTTTCACTGC |
| AYR1-SG-F | TCAATATACAACTTCCCTGAGTTTTAGAGCTAGAAATAGCAAGTTAAAATA |
| ATF2-F | GCGGGATAATGAGTAAAC |
| ATF2K-R | GCAGTATCGCAATTAACCTGGACAATTT |
| ATF2K-F | CAGGTTAATTGCGATACTGCCGTAGCGG |
| ATF2CX-R | CTGGACAATTGTTCTGAT |
| ATF2-SG-F | AAAATTGTCCAGGTTAATTAGTTTTAGAGCTAGAAATAGCAA |
| ATF2-SG-R | TAATTAACCTGGACAATTTTGATCATTTATCTTTCACTGCGG |
| HISAR-R | TCGCCTCGAGAGGAACAGTCATGTCTAAGGCG |
| ADH2T-F | GACTGTTCCTCTCGAGGCGAATTTCTTATGAT |
| URA3Lac-F | ACCATACCACCTTTTCAATTCATCATTTTT |
| LAC3p-R | ATATGATCCGCCTGGGTAATAACTCGATAT |
| PLACi-F | ATTACCCAGGCGGATCATATATGCGGTGTGAAATACCG |
| PLACi-R | AATTGAAAAGGTGGTATGGTGCACTCTCAGTACAATCT |
| QALG9-F | ATTTTACGGGAAGCTTGCTC |
| QAGL9-R | AGTAGTACCATGGCTCAACC |
| QHSD-F | ACGTCTCGGTCGTCATCCACA |
| QHSD-R | CCAACAGTAGCTGGGTACCTT |
| HXT1-F | AAATGCCCACCTGACCATCC |
| HXT1--R | GCCCCAAGATGCAGTACCAG |
| SUL1-F | ACAATCTAACTGCGAAATCGG |
| SUL1-R | GCCACTAAATCAGCATAGCC |
| RRI1-F | ACAGATATTCCGAGTTACACC |
| RRI1-R | TTCAATATTTCCTCCACGAAC |
| LIF1-F | TCGCTGAACTTACCTTGCAT |
| LIF1-R | CCGGTAAGAATCGTTAACACA |
| TPO2-F | CCTTAGCTCCAAATCTAGGTTG |
| TPO2-R | AACATATCGGCAATAGACCC |
| SSA3-F | CGACAGATGCCAAGCACTTCC |
| SSA3-R | CCTCAGGCGTAAATGTTTTCGT |
| PHM8-F | TATTGCCACTACGACAGACCC |
| PHM8-R | AAAGCTCGACAACCCACT |
| YEH1-F | GCCACCCATTTTGATGCTAC |
| YEH1-R | ACCATATGTCGTAACCGGAT |
| CTR3-F | AAATTTGCTGGTTCTTGCAT |
| CTR3-R | TAGCTCTACGTCAAATTGCCTT |
| ADE12-F | CCCAAACTGCCAAAACCTT |
| ADE12-R | TCCTTGCGTTCTTCAAACCT |
| SUI2-F | TCGTCATGGTTAACGTCCAG |
| SUI2-R | TCTACGGGACAATTCACT |
| THI2-F | AAATCGAAACCATTGTGCCAGA |
| THI2-R | ACGCTGTCTTCGCAGTCAC |

Table S4.

Four-parameter logistic (4PL) dose–response fitting parameters for EGFP fluorescence in progesterone-responsive *Saccharomyces cerevisiae* strains (S067–S079).

| Strain | Bottom (a.u.) | Top (a.u.) | Span (a.u.) | IC₅₀ (μM) | HillSlope | R² |
| --- | --- | --- | --- | --- | --- | --- |
| S067 | 510.6 | 26318 | 25807 | 26.13 | 3.018 | 0.998 |
| S068 | 669.2 | 29466 | 28797 | 21.83 | 2.923 | 0.999 |
| S077 | 477.1 | 1945 | 1468 | 24.32 | 2.045 | 0.991 |
| S078 | 355 | 1149 | 794.3 | 28.99 | 3.486 | 0.982 |
| S079 | 385.5 | 864.8 | 479.3 | 25.05 | — | 0.976 |

Note: Measurements were performed in SD/-Leu medium supplemented with 4% HP-β-CD at 30 °C and 250 rpm. Strains S077–S079 carried LacI–LacO modules for tunable repression, whereas S067 and S068 lacked regulatory control. Bottom and Top represent minimum and maximum fluorescence intensities at OD600 = 1 (normalized, a.u.), respectively; Span = Top − Bottom indicates the dynamic range; IC₅₀ is the progesterone concentration at half-maximal induction (μM); HillSlope reflects the steepness of the dose–response curve; R² indicates goodness of fit. HillSlope for S079 could not be reliably determined due to unstable fitting. Fluorescence values were normalized to the fluorescence intensity at OD600 = 1.

Table S5.

4PL fitting parameters of EGFP fluorescence dose–response curves under different IPTG concentrations in S077 strain.

| IPTG (mM) | Bottom (a.u.) | Top (a.u.) | Span (a.u.) | IC₅₀ (μM) | HillSlope | R² |
| --- | --- | --- | --- | --- | --- | --- |
| 0 | 476.9 | 1944 | 1467 | 24.32 | 2.05 | 0.9893 |
| 0.1 | 482 | 1859 | 1377 | 15.89 | 2.407 | 0.9893 |
| 0.5 | 497.7 | 2074 | 1577 | 12.78 | 1.911 | 0.9827 |
| 1 | 516.6 | 2988 | 2472 | 20.4 | 1.465 | 0.984 |
| 5 | 598.2 | 7790 | 7192 | 40.63 | 1.225 | 0.9932 |
| 10 | 561.8 | 13941 | 13379 | 42.86 | 1.39 | 0.996 |
| 20 | 640.4 | 17281 | 16641 | 34.42 | 1.648 | 0.997 |
| 30 | 722.4 | 18110 | 17388 | 30.2 | 1.91 | 0.9974 |

Note: Measurements were performed in triplicate after 12–14 h incubation in SD/-Leu medium supplemented with 4% HP-β-CD at 30 °C and 250 rpm. Bottom and Top represent minimum and maximum fluorescence intensities at OD600 = 1 (normalized, a.u.), respectively; Span = Top − Bottom indicates the dynamic range of the response; IC₅₀ is the progesterone concentration at half-maximal induction (μM); HillSlope reflects the steepness of the dose–response curve; R² indicates goodness of fit. Fluorescence values were normalized to the fluorescence intensity at OD600 = 1.

Table S6.

Upregulated genes in mutant S092 compared with wild-type S081.

| Gene Name | log_2_FC | padj | Function Category |
| --- | --- | --- | --- |
| HXT1 | 4.601 | 9.11 × 10^-51^ | Transporter / Membrane |
| HXT2 | 2.342 | 8.85 × 10^-11^ | Transporter / Membrane |
| PHO89 | 3.102 | 5.30 × 10^-16^ | Transporter |
| PTR2 | 2.423 | 1.99 × 10^-23^ | Transporter |
| FIT1 | 2.132 | 3.56 × 10^-14^ | Transporter |
| CIS3 | 2.563 | 7.78 × 10^-23^ | Membrane |
| TOH1 | 2.098 | 4.58 × 10^-14^ | Membrane |
| FRM2 | 2.461 | 4.41 × 10^-21^ | Oxidoreductase |
| BDH1 | 2.953 | 6.07 × 10^-19^ | Oxidoreductase |
| ADH5 | 2.239 | 8.82 × 10^-15^ | Oxidoreductase |
| GDH3 | 2.005 | 4.20 × 10^-12^ | Oxidoreductase |
| ARO8 | 2.029 | 6.00 × 10^-14^ | Oxidoreductase |
| ARO9 | 2.072 | 3.21 × 10^-14^ | Oxidoreductase |

Note: Genes with log_2_ fold change > 2 and adjusted p-value (padj) < 0.001 are listed. Function categories of the listed genes include membrane/transporters and oxidoreductases, suggesting potential mechanisms that contribute to the enhanced progesterone production observed in S092.
